# Supplementary material for: Associations in cell type-specific hydroxymethylation and transcriptional alterations of pediatric central nervous system tumors
Source: Nat Commun. 2024 Apr 30;15:3635. doi: 10.1038/s41467-024-47943-9 (PMC11061294; doi:10.1038/s41467-024-47943-9)
Supplement: Supplementary file 1 — Supplementary Information [file 41467_2024_47943_MOESM1_ESM.pdf]

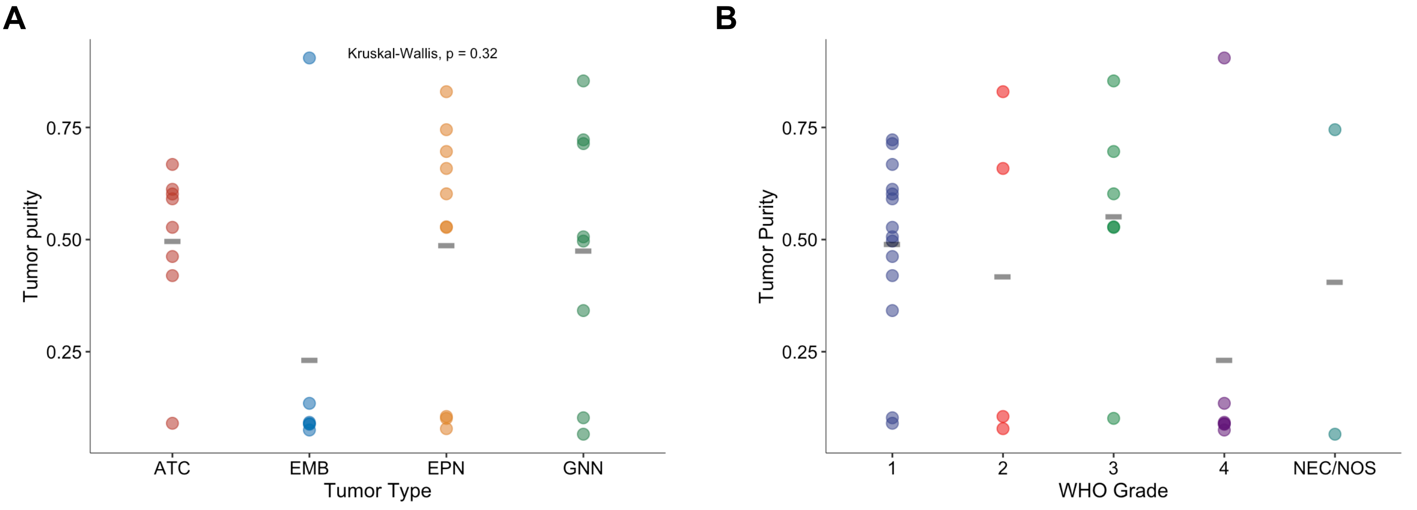

Supplementary Figure 1. Tumor purity differences by **A)** tumor type and **B)** grade.

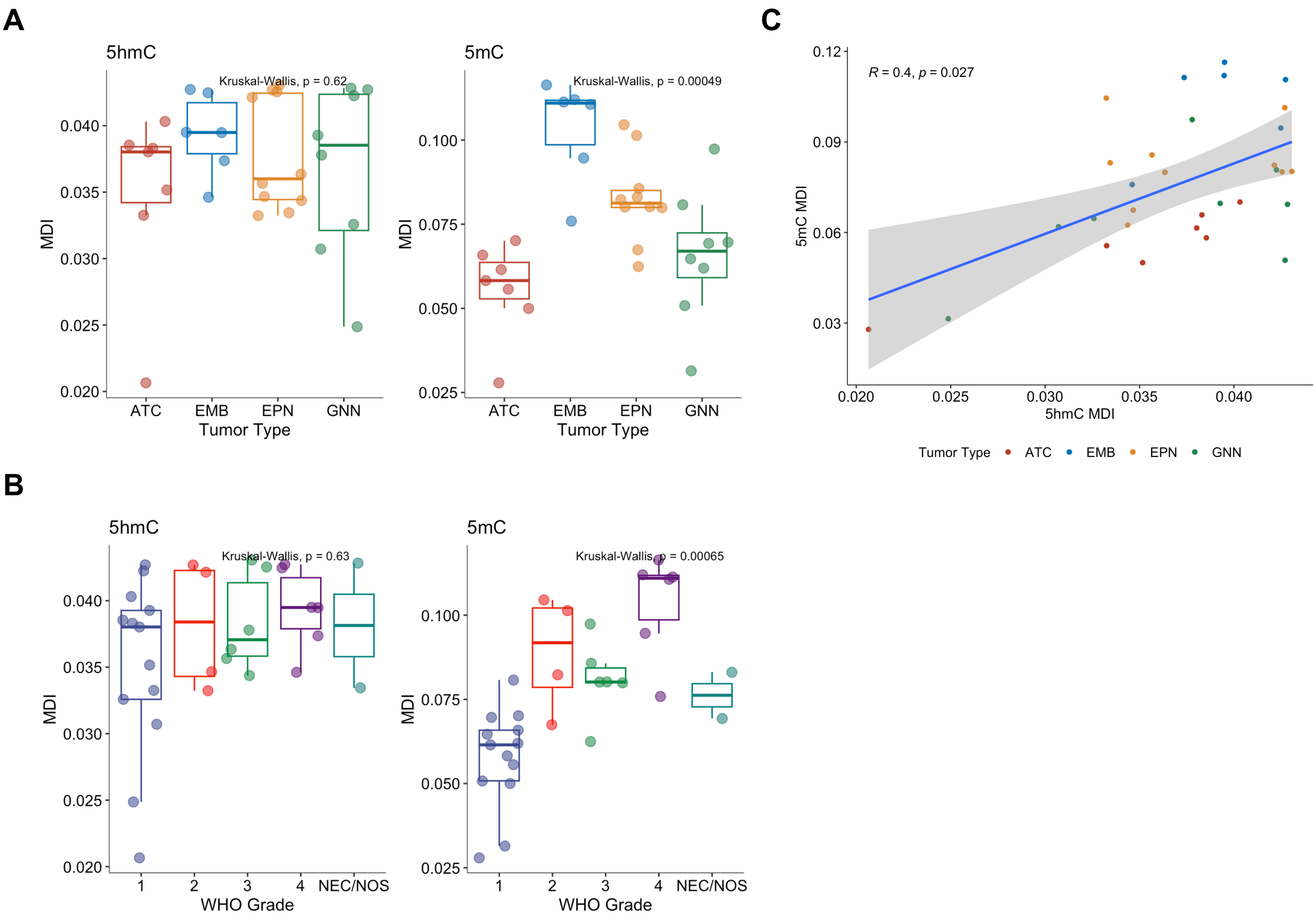

Supplementary Figure 2. Methylation dysregulation index (MDI) from 5-hmC and 5-mC by **A)** tumor type and **B)** grade without outliers. Differences in MDI were calculated using the Kruskal-Wallis test. **C)** Correlation between 5-hmC MDI and 5-mC MDI calculated using Spearman rank correlation. The linear regression line is indicated by the blue line. 95% confidence interval of the linear regression line indicated by gray bands. Color of each point indicates tumor type. In the boxplots of **A)** and **C)**, the low ends of the segment indicate the minimum and the high ends of the segment indicate the maximum. Lower bounds of the box indicate the 25th percentile and the higher bounds of the box indicate the 75th percentile. Segment in the middle is the median.

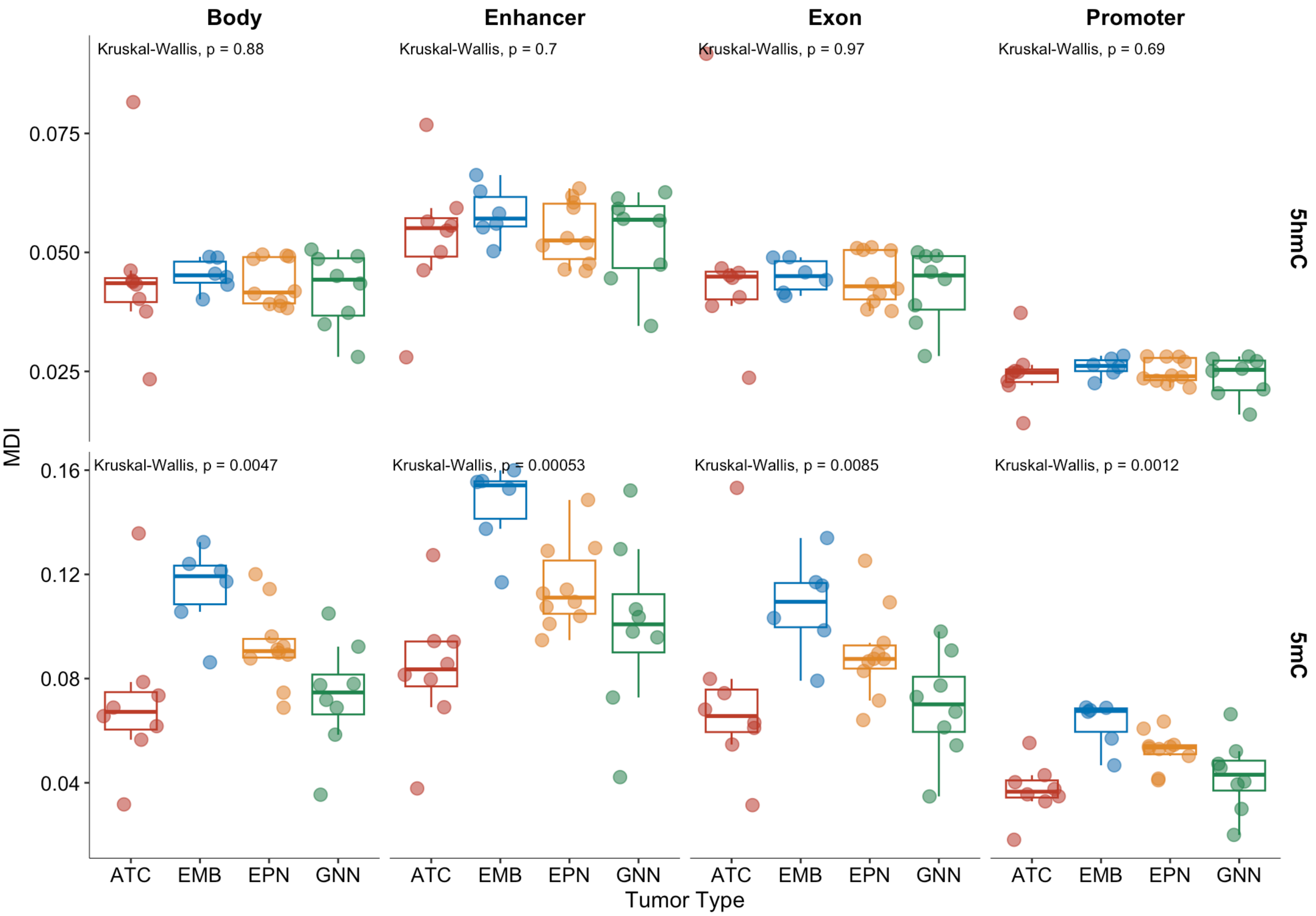

Supplementary Figure 3. Methylation dysregulation index (MDI) at each genomic context for 5-hmC and 5-mC. In the boxplots, the low ends of the segment indicate the minimum and the high ends of the segment indicate the maximum. Lower bounds of the box indicate the 25th percentile and the higher bounds of the box indicate the 75th percentile. Segment in the middle is the median. The distributions of MDI among the tumor types were compared using the Kruskal-Wallis test.

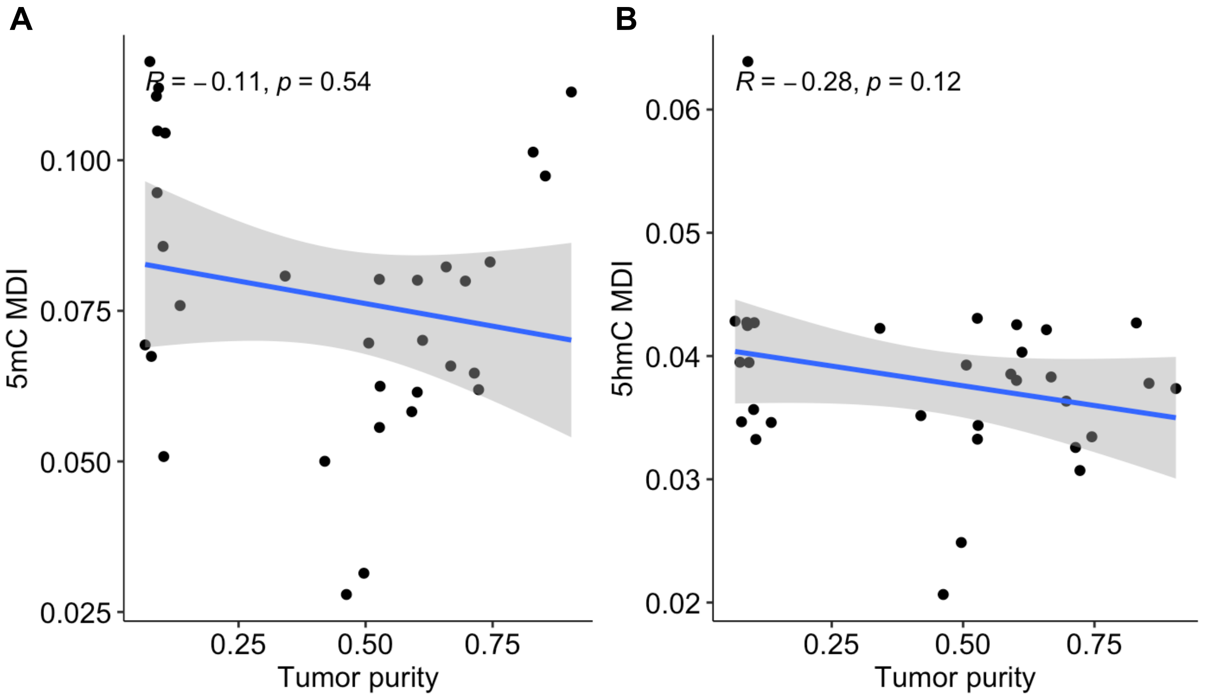

Supplementary Figure 4. Tumor purity is not associated with MDI. Correlation between tumor purity and **A)** 5-mC MDI and **B)** 5-hmC MDI calculated using Spearman rank correlation. The linear regression line is indicated by the blue line. 95% confidence interval of the linear regression line indicated by gray bands.

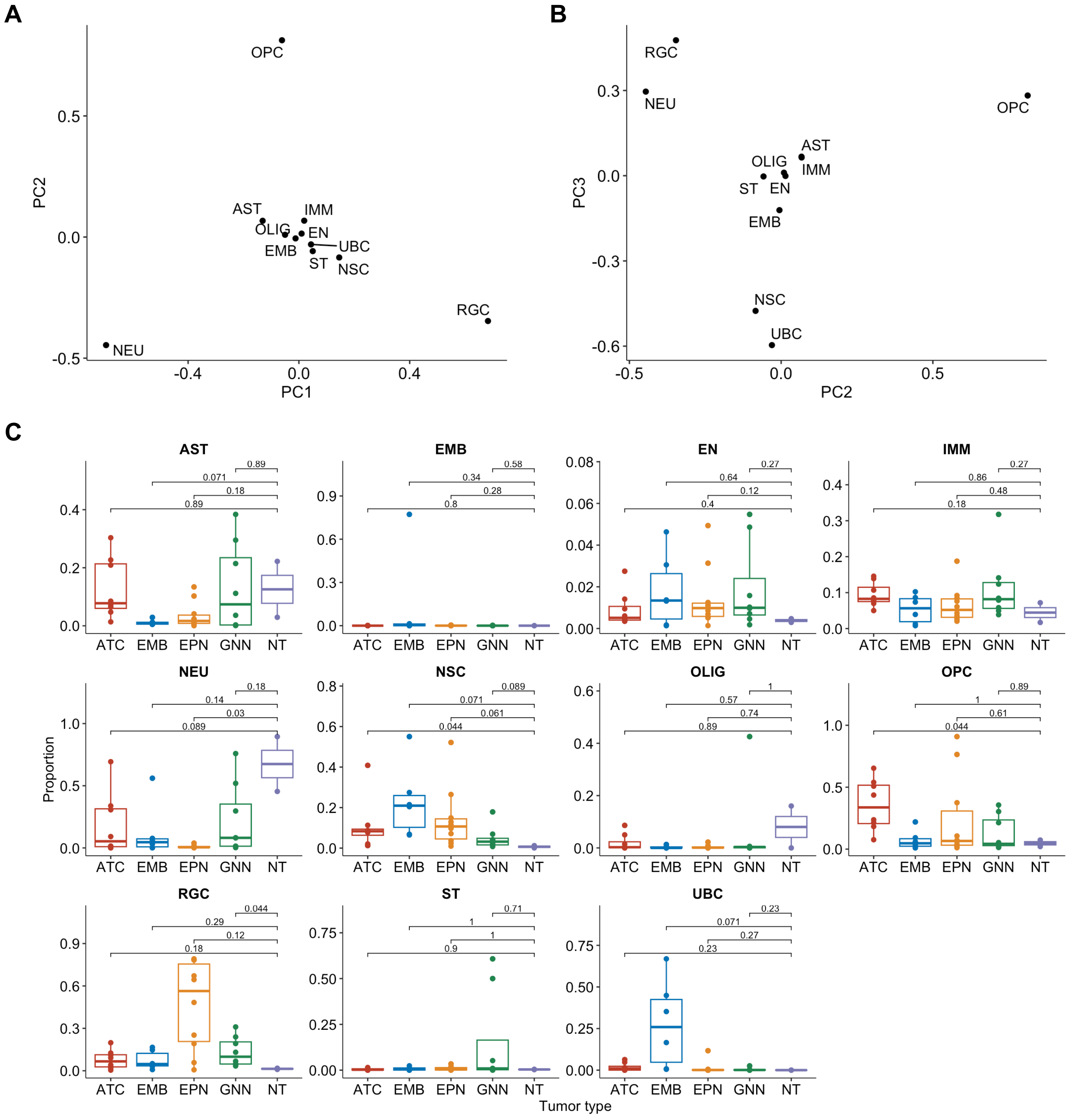

Supplementary Figure 5. A, B) Results from principal component analysis of cell type proportions from single nuclei RNA-seq of pediatric central nervous system tumors and non-tumor pediatric brain tissue. **C)** Comparison of each tumor type's proportions per cell type against the proportions found in non-tumor pediatric brain tissue. Each point indicates a sample. Differences in proportions are calculated using two-sided Wilcoxon signed-rank test. In the boxplots, the low ends of the segment indicate the minimum and the high ends of the segment indicate the maximum. Lower bounds of the box indicate the 25th percentile and the higher bounds of the box indicate the 75th percentile. Segment in the middle is the median.

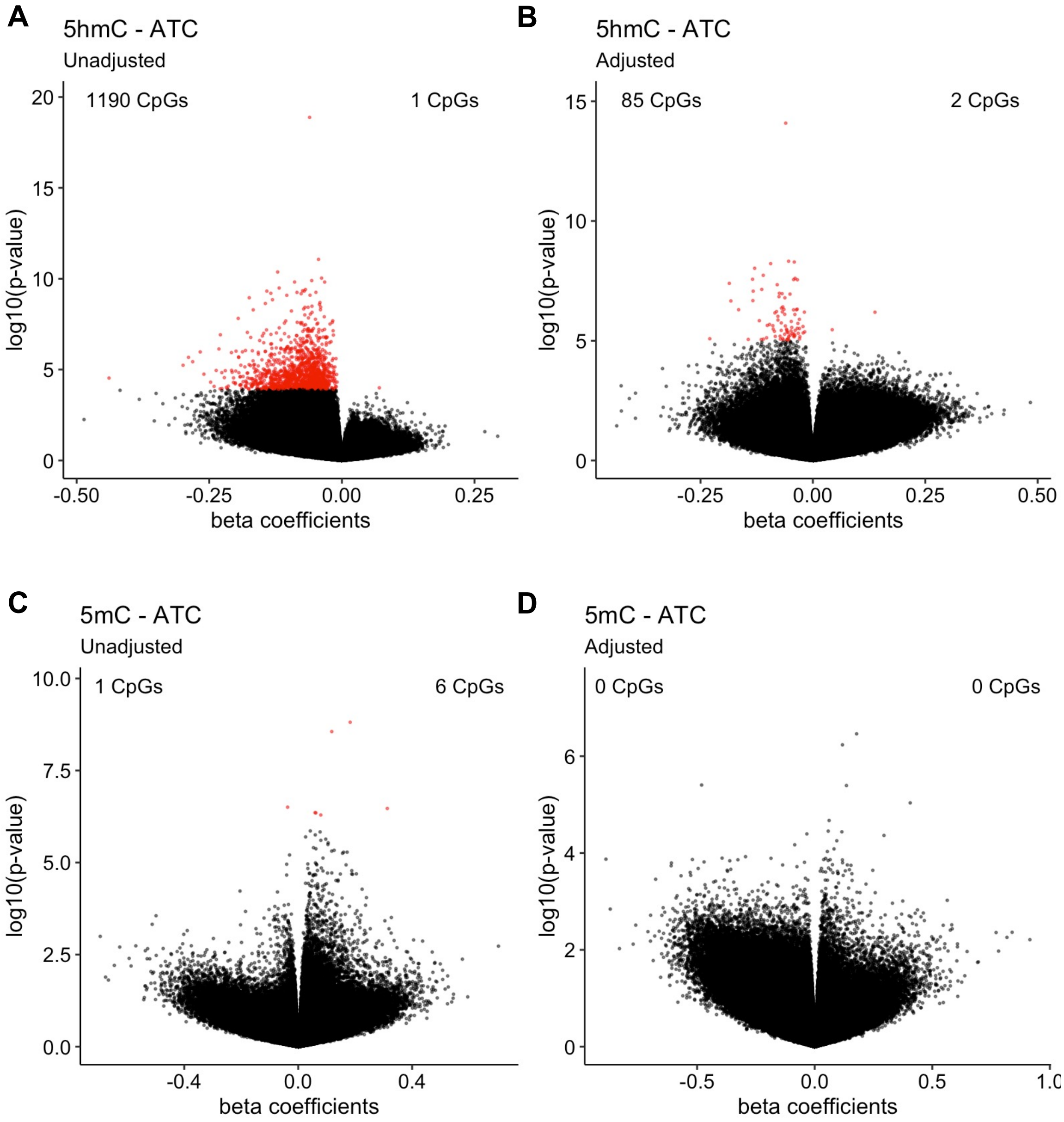

Supplementary Figure 6. Volcano plots of differential **A)** 5-hmC CpGs and **C)** 5-mC CpGs in astrocytoma in the cell type proportion unadjusted model. Volcano plots of differential **B)** 5-hmC CpGs and **D)** 5-mC CpGs in astrocytoma in the cell type proportion adjusted model. Labeled # of CpGs on the left of each plot are CpGs with decreased methylation in tumors compared to non-tumor tissue. Labeled # of CpGs on the right of each volcano plot are CpGs with increased methylation in tumors compared to non-tumor tissue. Red points indicate statistically significant differential CpGs under the q-value < 0.05 threshold.

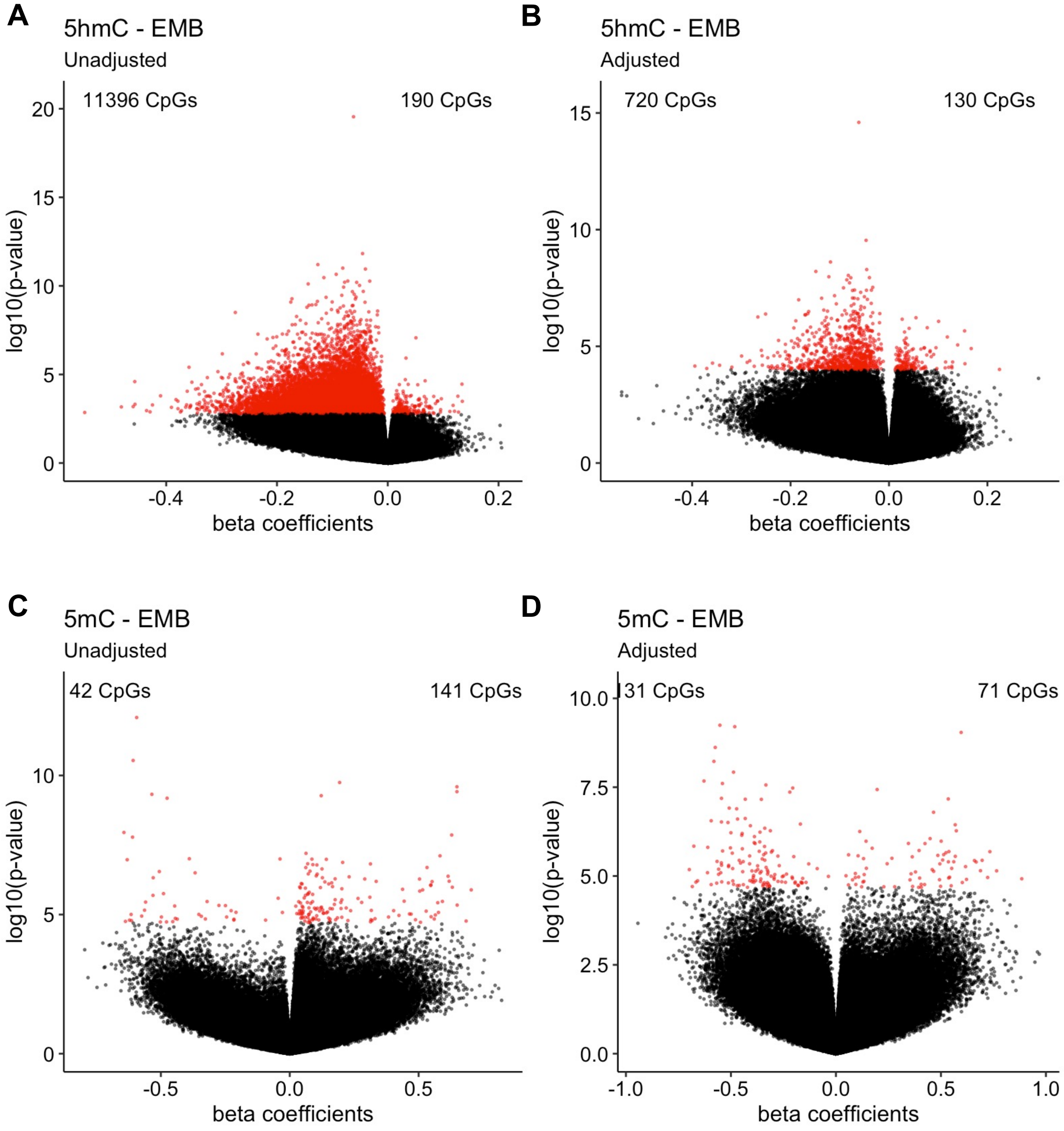

Supplementary Figure 7. Volcano plots of differential **A)** 5-hmC CpGs and **C)** 5-mC CpGs in embryonal tumors in the cell type proportion unadjusted model. Volcano plots of differential **B)** 5-hmC CpGs and **D)** 5-mC CpGs in astrocytoma in the cell type proportion adjusted model. Labeled # of CpGs on the left of each plot are CpGs with decreased methylation in tumors compared to non-tumor tissue. Labeled # of CpGs on the right of each volcano plot are CpGs with increased methylation in tumors compared to non-tumor tissue. Red points indicate statistically significant differential CpGs under the q-value < 0.05 threshold.

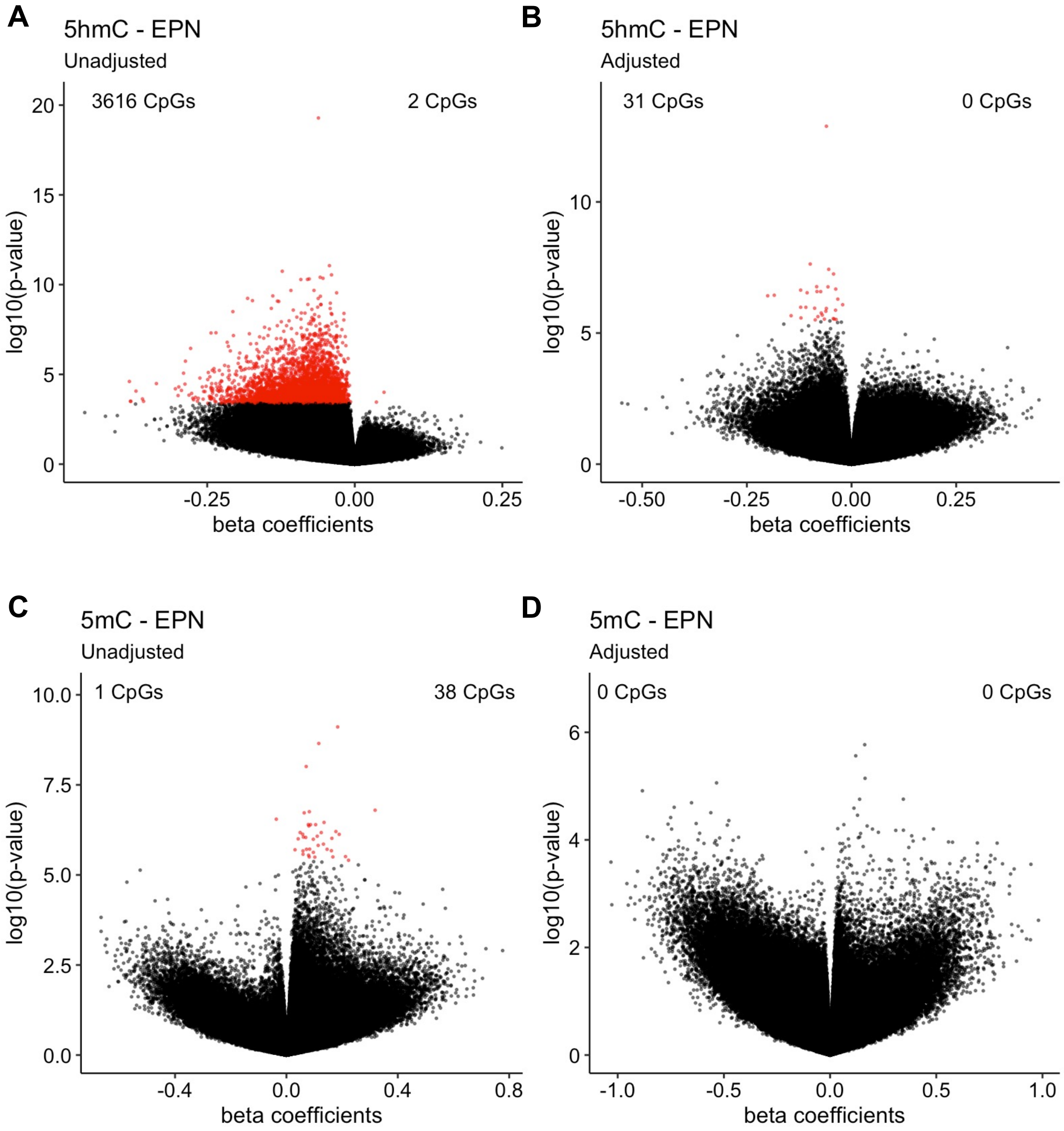

Supplementary Figure 8. Volcano plots of differential **A)** 5-hmC CpGs and **C)** 5-mC CpGs in ependymoma in the cell type proportion unadjusted model. Volcano plots of differential **B)** 5-hmC CpGs and **D)** 5-mC CpGs in astrocytoma in the cell type proportion adjusted model. Labeled # of CpGs on the left of each plot are CpGs with decreased methylation in tumors compared to non-tumor tissue. Labeled # of CpGs on the right of each volcano plot are CpGs with increased methylation in tumors compared to non-tumor tissue. Red points indicate statistically significant differential CpGs under the q-value < 0.05 threshold.

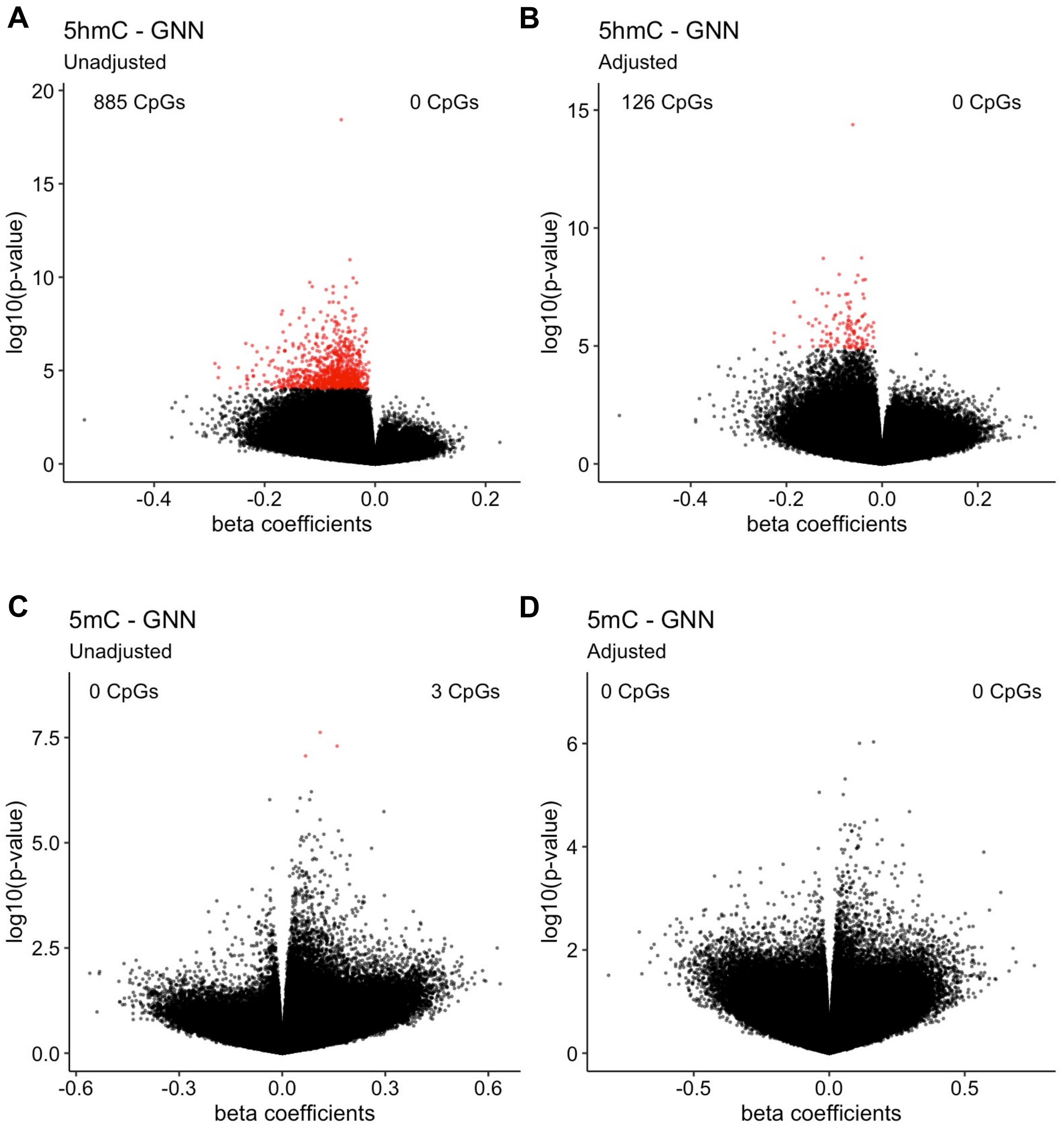

Supplementary Figure 9. Volcano plots of differential **A)** 5-hmC CpGs and **C)** 5-mC CpGs in glioneuronal/neuronal tumors in the cell type proportion unadjusted model. Volcano plots of differential **B)** 5-hmC CpGs and **D)** 5-mC CpGs in astrocytoma in the cell type proportion adjusted model. Labeled # of CpGs on the left of each plot are CpGs with decreased methylation in tumors compared to non-tumor tissue. Labeled # of CpGs on the right of each volcano plot are CpGs with increased methylation in tumors compared to non-tumor tissue. Red points indicate statistically significant differential CpGs under the q-value < 0.05 threshold.

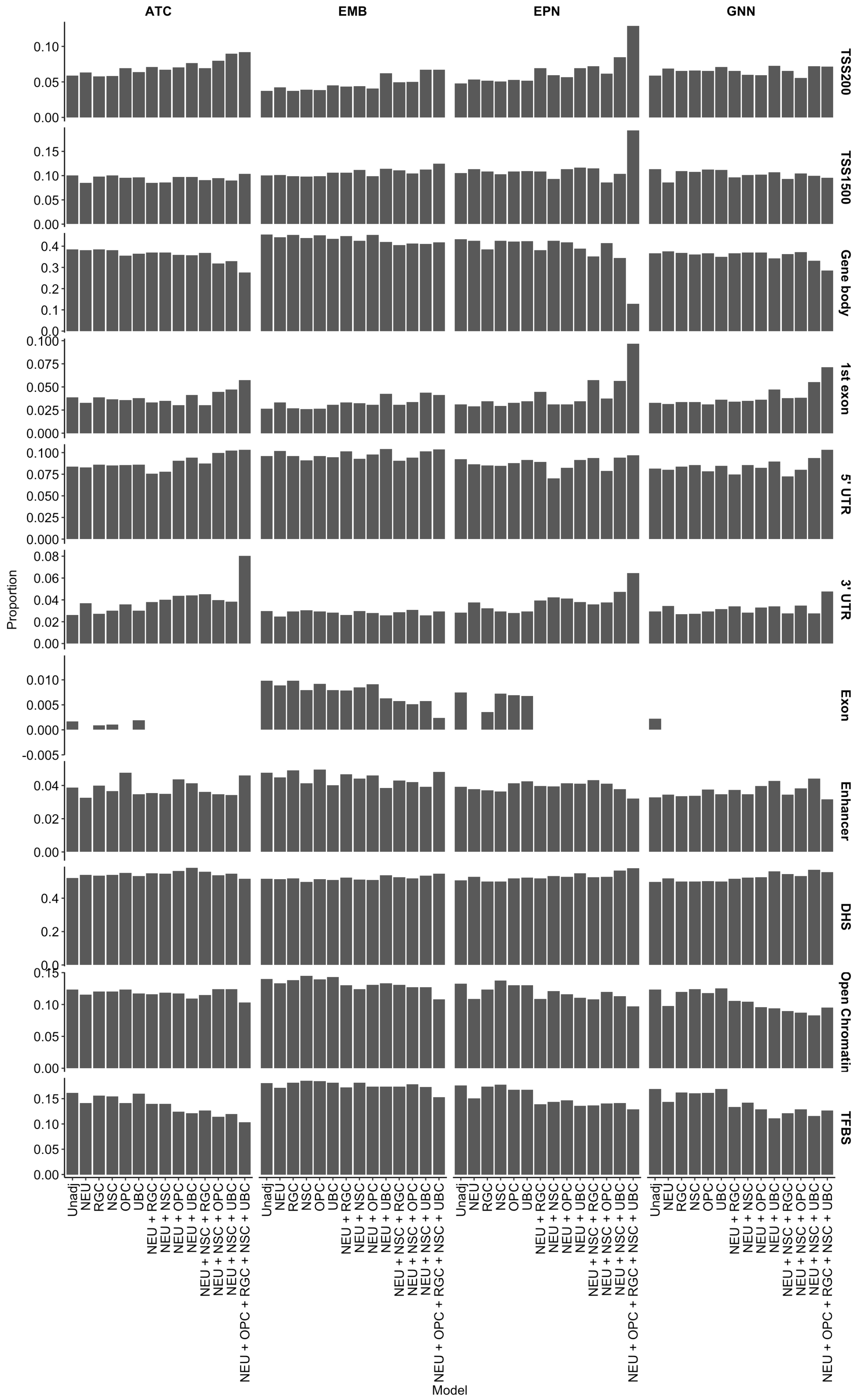

Supplementary Figure 10. Proportion of dhmCpGs at each genomic context over each model with various cell type proportions included for each tumor type.

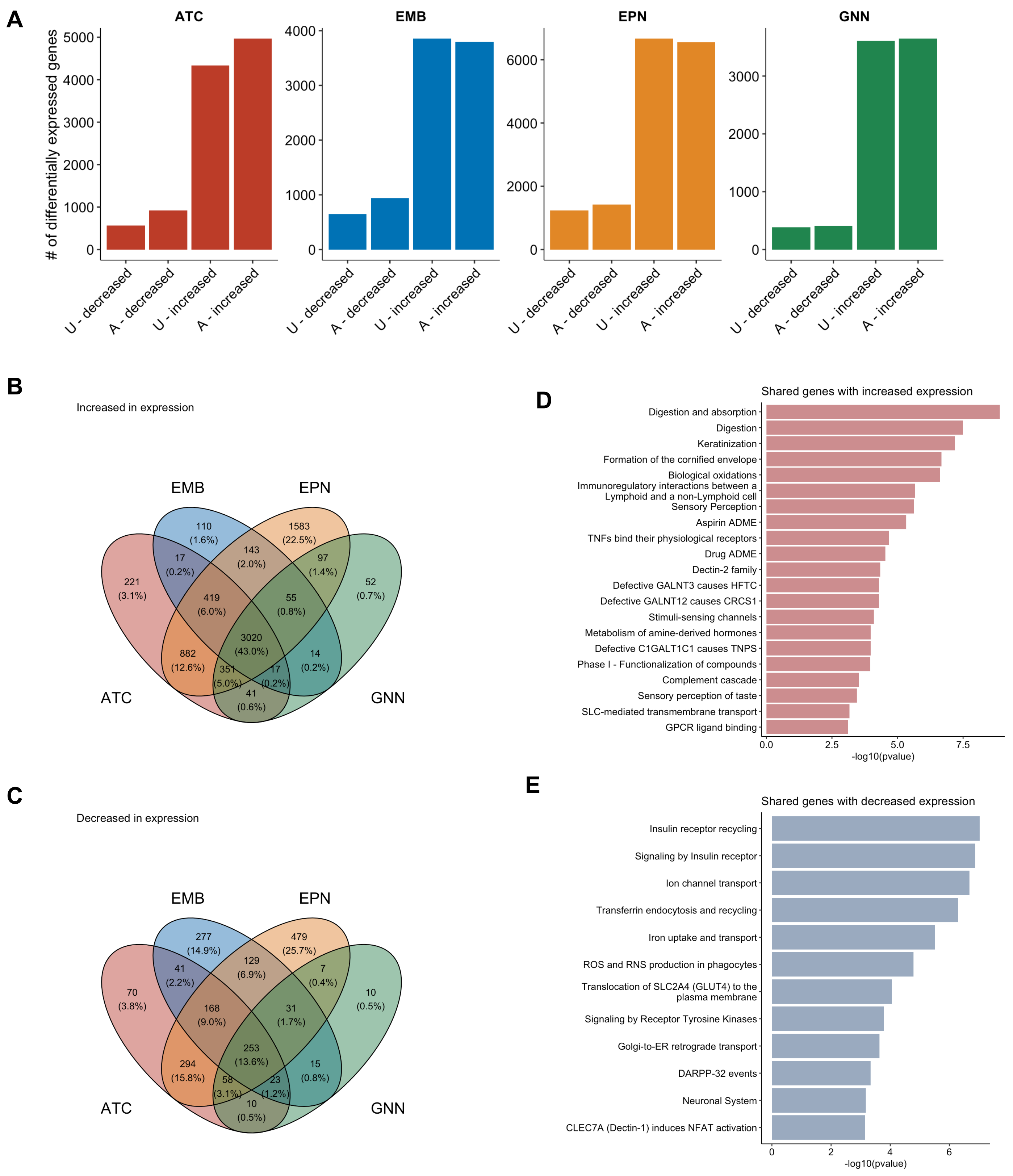

Supplementary Figure 11. A) Number of differentially expressed genes unadjusted and adjusted for cell type proportions for each tumor type. Genes under adjusted p-value < 0.05 were considered to be significantly differentially expressed. Venn diagram of the genes with significant **B)** increased and **C)** decreased expression in the tumor types. **D)** Pathways associated with shared genes with increased expression in the tumors. Only pathways under q-value < 0.05 are shown. **E)** Pathways associated with shared genes with decreased expression in the tumors. Only pathways under q-value < 0.05 are shown.

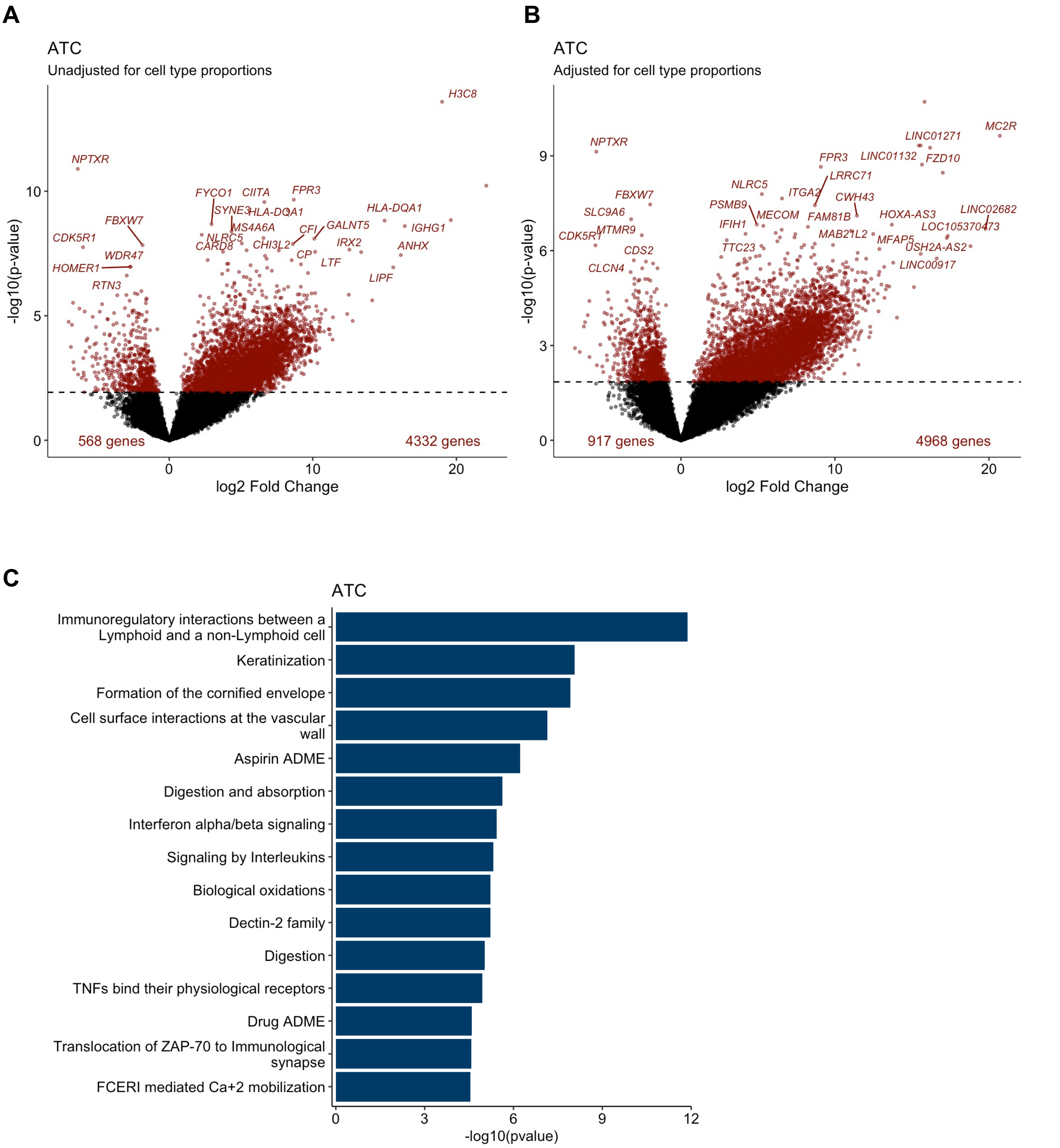

Supplementary Figure 12. Volcano plot of differential expression test in the **A)** cell type proportion unadjusted and **B)** cell type proportion adjusted model comparing astrocytoma and non-tumor brain tissue. **C)** Pathways associated with the differential expression in astrocytoma.

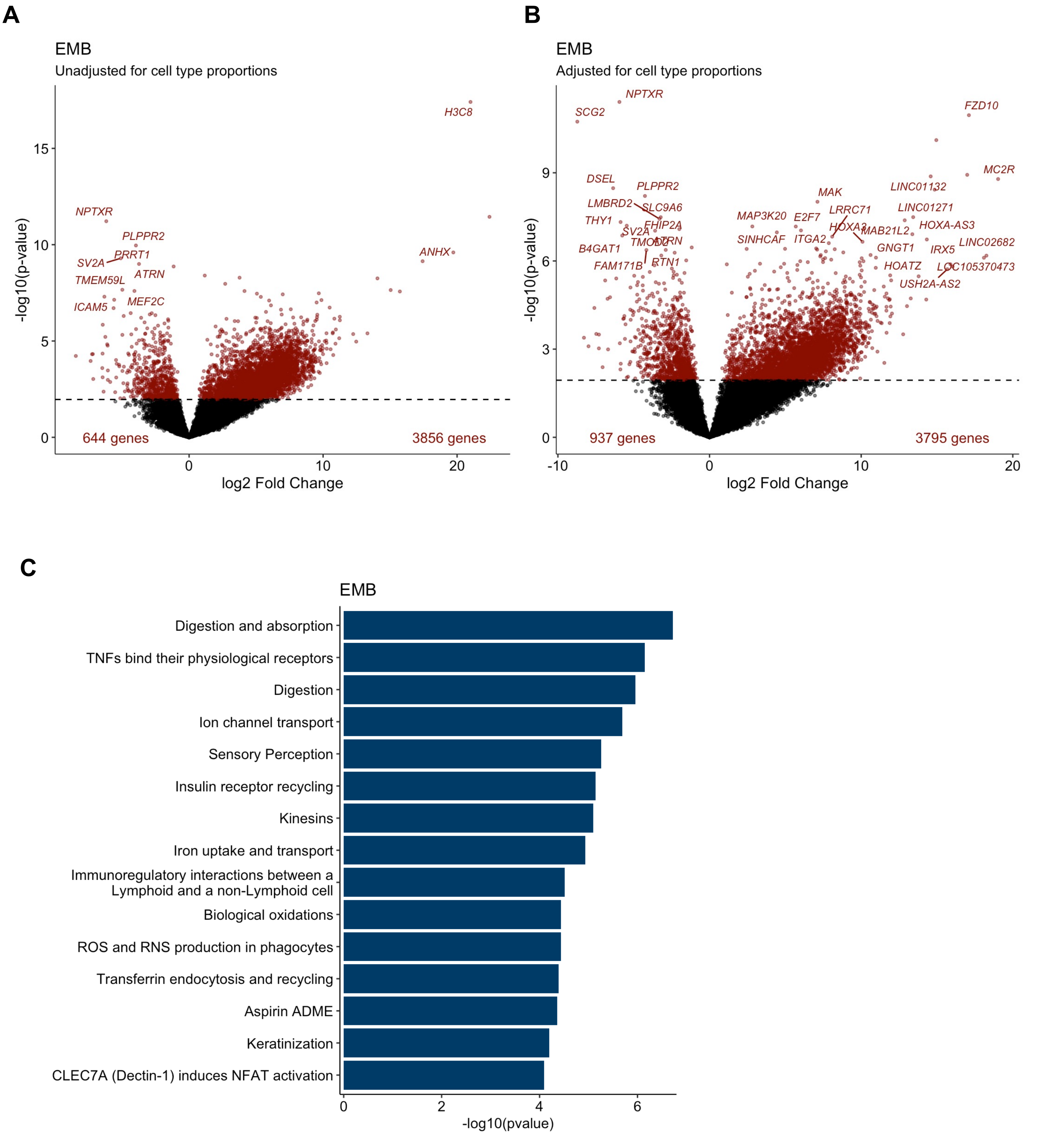

Supplementary Figure 13. Volcano plot of differential expression test in the **A)** cell type proportion unadjusted and **B)** cell type proportion adjusted model comparing embryonal tumors and non-tumor brain tissue. **C)** Pathways associated with the differential expression in embryonal tumors.

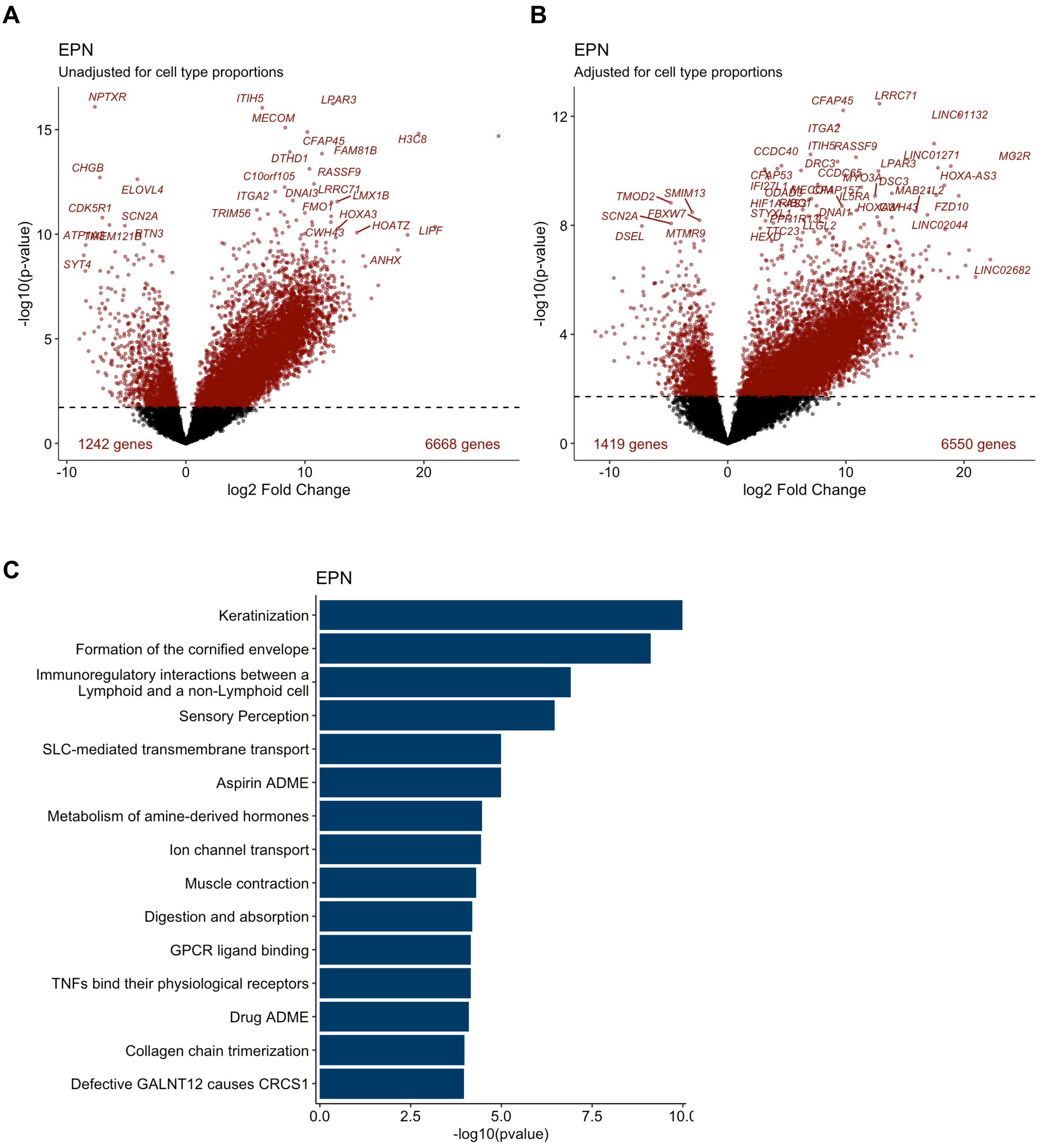

Supplementary Figure 14. Volcano plot of differential expression test in the **A)** cell type proportion unadjusted and **B)** cell type proportion adjusted model comparing ependymoma and non-tumor brain tissue. **C)** Pathways associated with the differential expression in ependymoma.

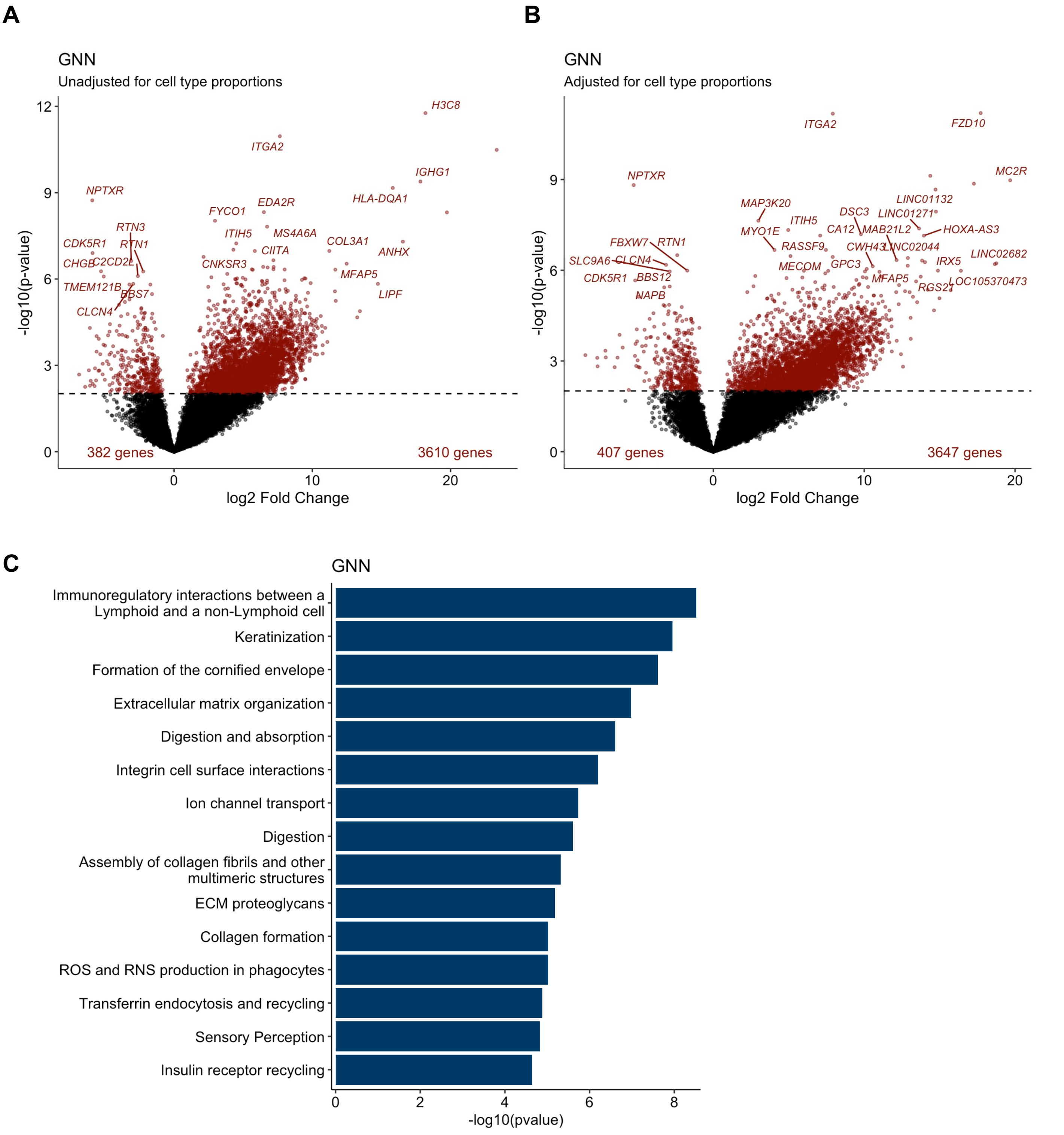

Supplementary Figure 15. Volcano plot of differential expression test in the **A)** cell type proportion unadjusted and **B)** cell type proportion adjusted model comparing glioneuronal/neuronal tumors and non-tumor brain tissue. **C)** Pathways associated with the differential expression in glioneuronal/neuronal tumors.

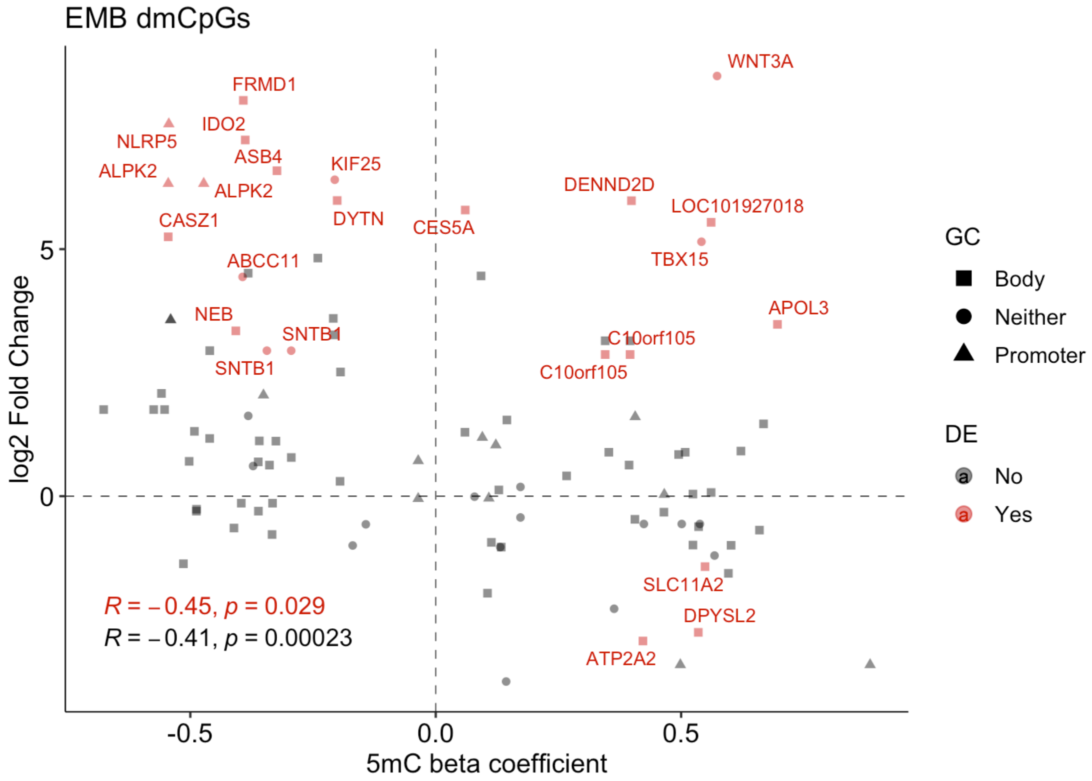

Supplementary Figure 16. Association between changes in 5-mC and gene expression for embryonal tumors. Red points indicate significantly differentially expressed genes. Shape of point indicates genomic context of CpG.

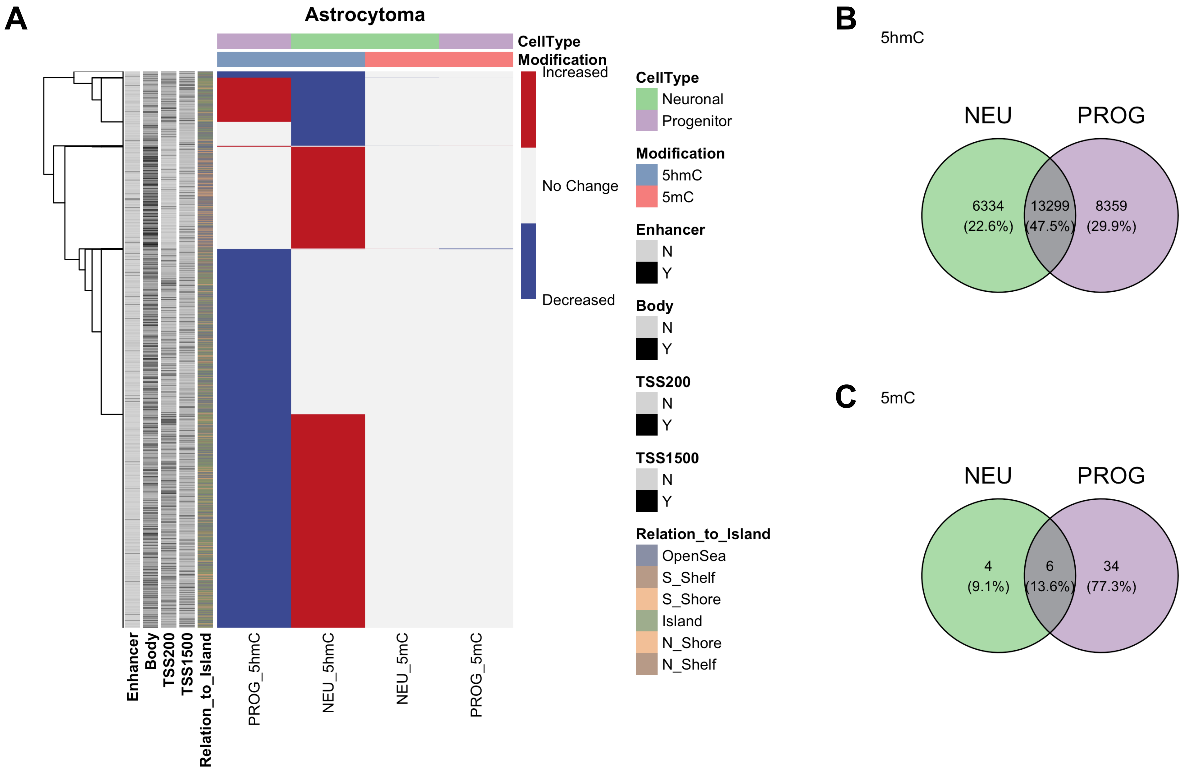

Supplementary Figure 17. A) Cell type-specific differentially hydroxymethylated and methylated CpGs in astrocytoma. Venn diagram of differentially **B)** hydroxymethylated and **C)** methylated CpGs in neuronal-like cell types (NEU) and progenitor-like cell types (PROG).

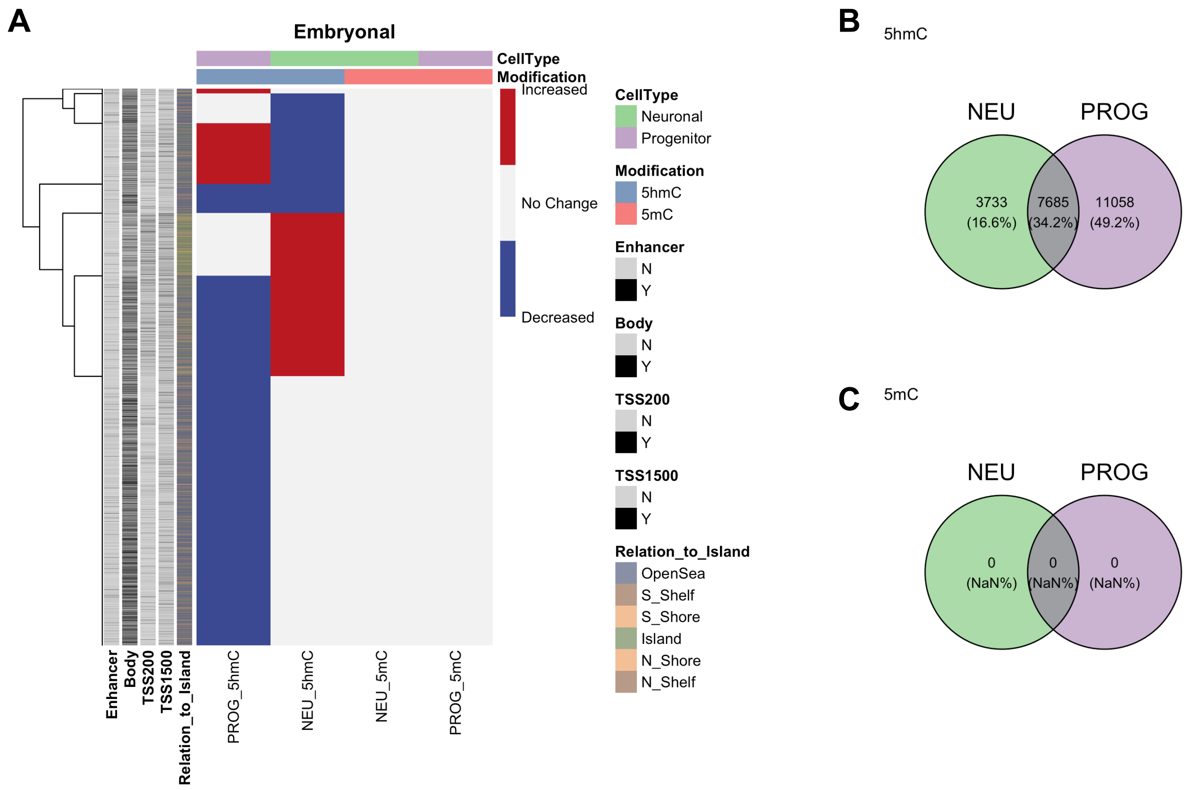

Supplementary Figure 18. A) Cell type-specific differentially hydroxymethylated and methylated CpGs in embryonal tumors. Venn diagram of differentially **B)** hydroxymethylated and **C)** methylated CpGs in neuronal-like cell types (NEU) and progenitor-like cell types (PROG).

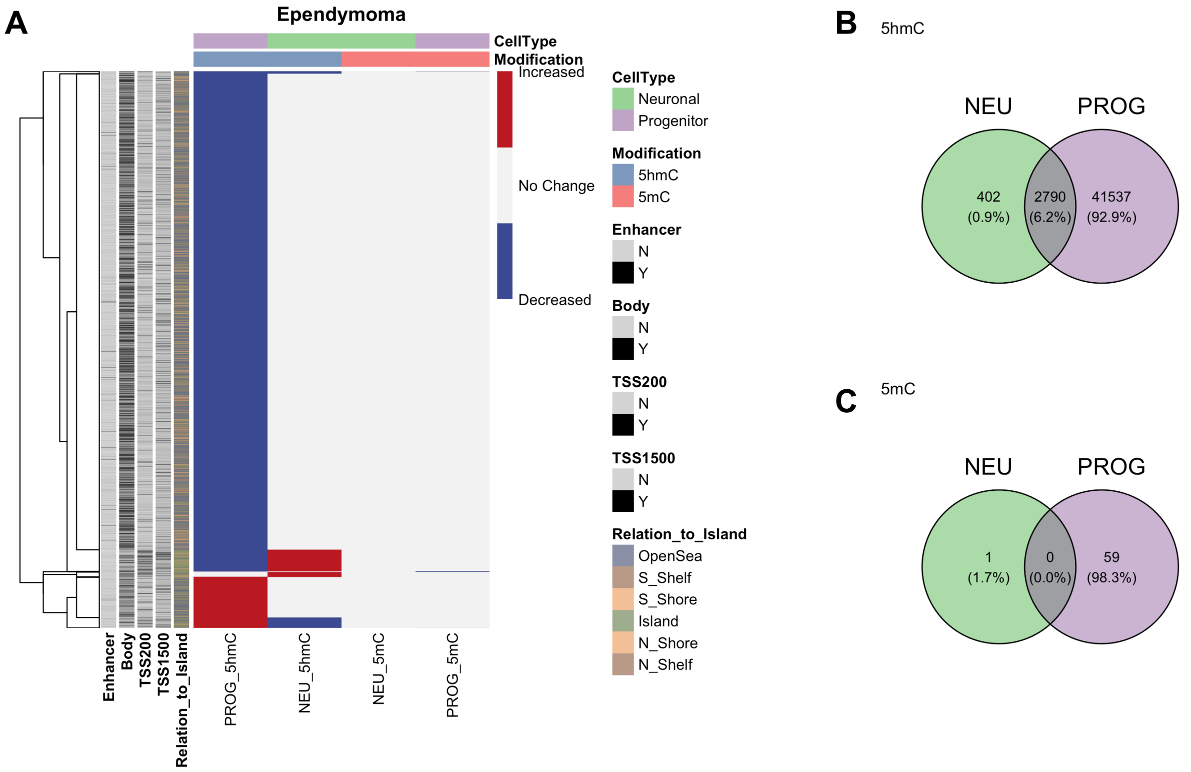

Supplementary Figure 19. A) Cell type-specific differentially hydroxymethylated and methylated CpGs in ependymoma. Venn diagram of differentially **B)** hydroxymethylated and **C)** methylated CpGs in neuronal-like cell types (NEU) and progenitor-like cell types (PROG).

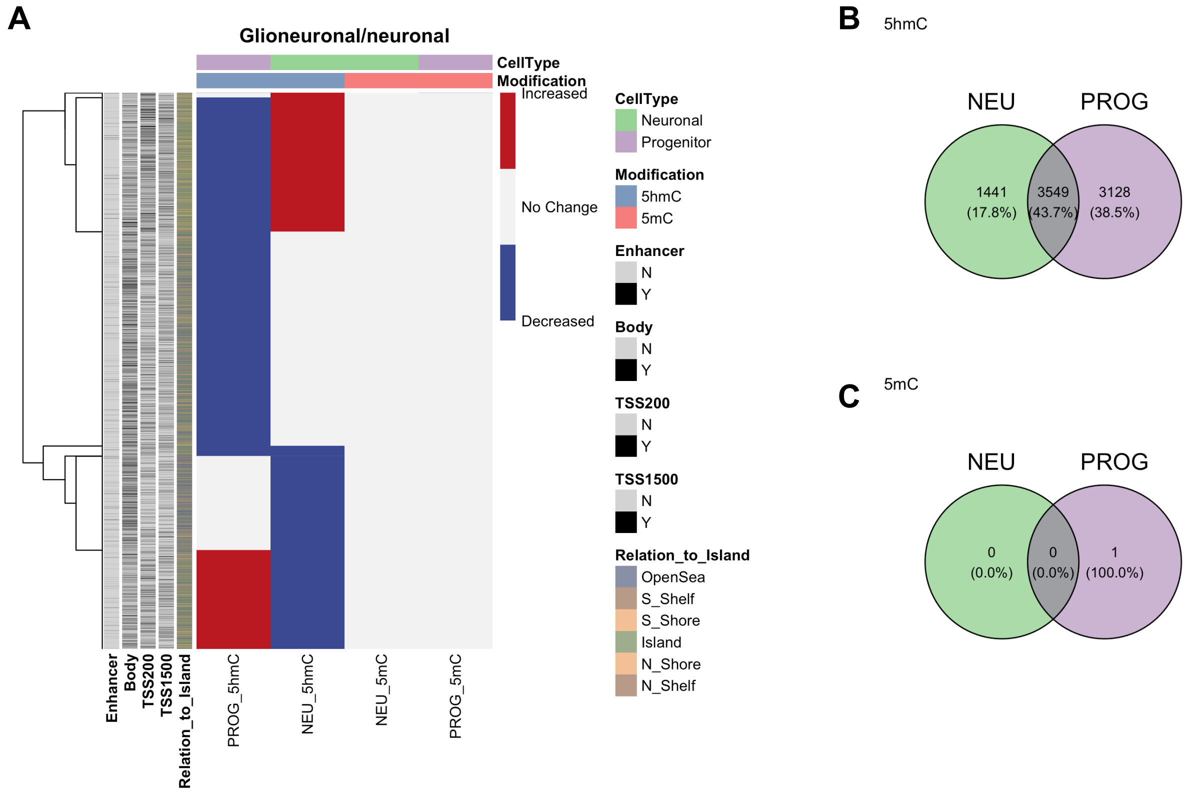

Supplementary Figure 20. A) Cell type-specific differentially hydroxymethylated and methylated CpGs in glioneuronal/neuronal tumors. Venn diagram of differentially **B)** hydroxymethylated and **C)** methylated CpGs in neuronal-like cell types (NEU) and progenitor-like cell types (PROG).

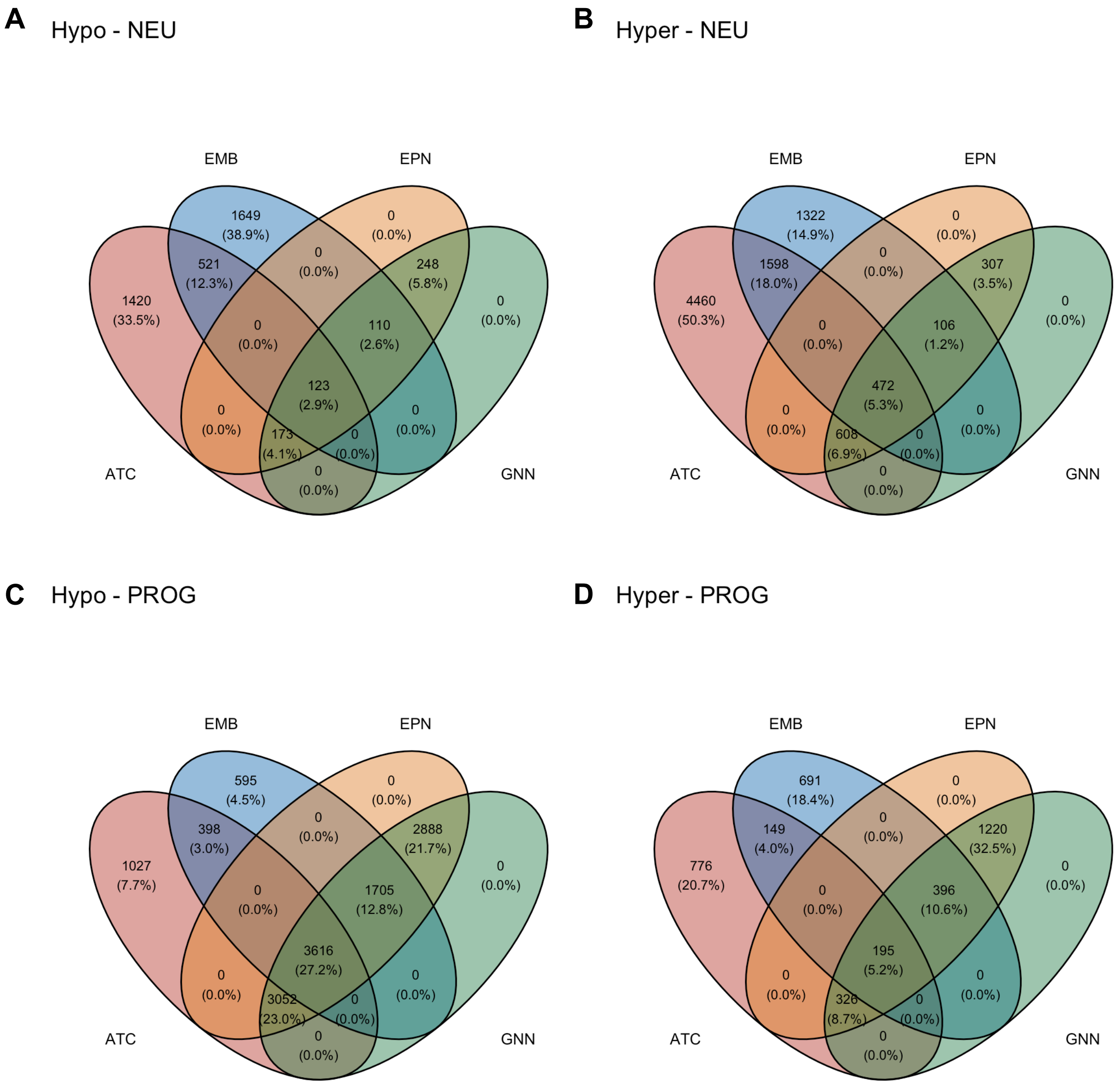

Supplementary Figure 21. Venn diagram of **A)** hypomethylated and **B)** hypermethylated CpGs in neuronal-like cell types across tumor types. Venn diagram of **C)** hypomethylated and **D)** hypermethylated CpGs in progenitor-like cell types across tumor types.

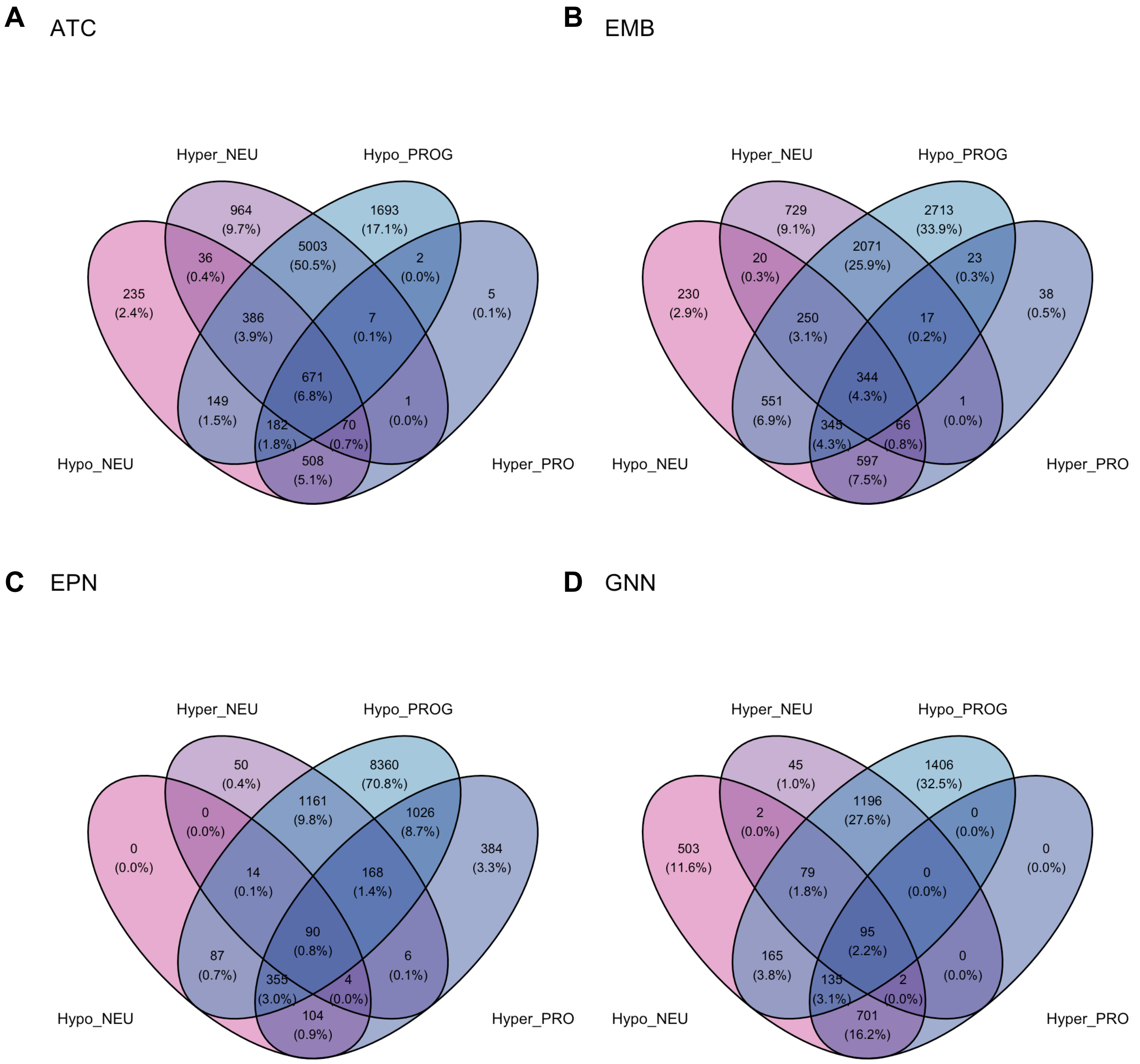

Supplementary Figure 22. Venn diagram of genes with differentially hydroxymethylated CpGs in **A)** astrocytomas, **B)** embryonal tumors, **C)** ependymomas, and **D)** glioneuronal/neuronal tumors.

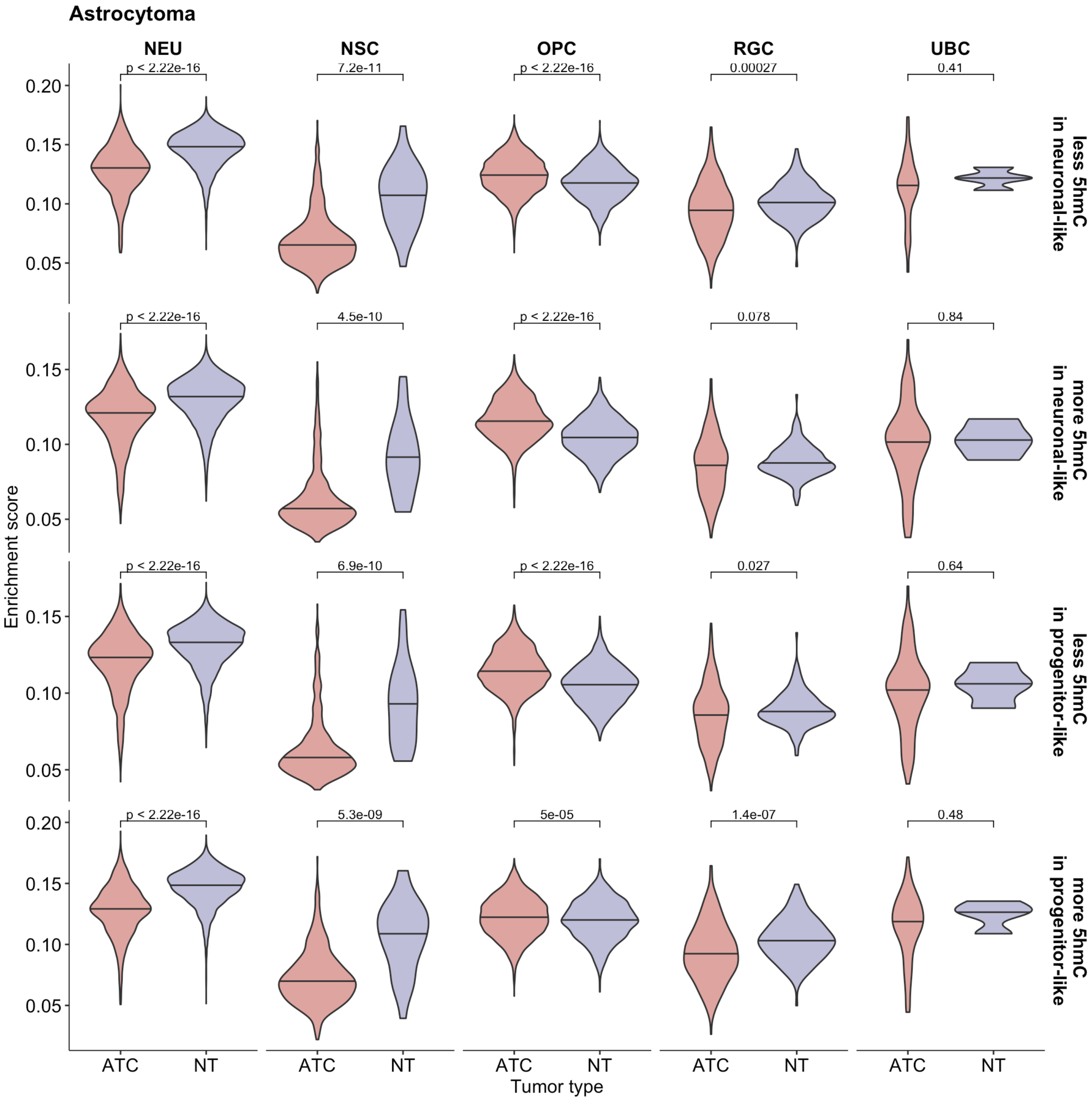

Supplementary Figure 23. Violin of enrichment scores of genes with differentially hydroxymethylated CpGs per cell type in astrocytoma and non-tumor brain tissue. Comparison between tumor and non-tumor was made with two-sided Wilcoxon rank test. Raw p-values are indicated for each comparison.

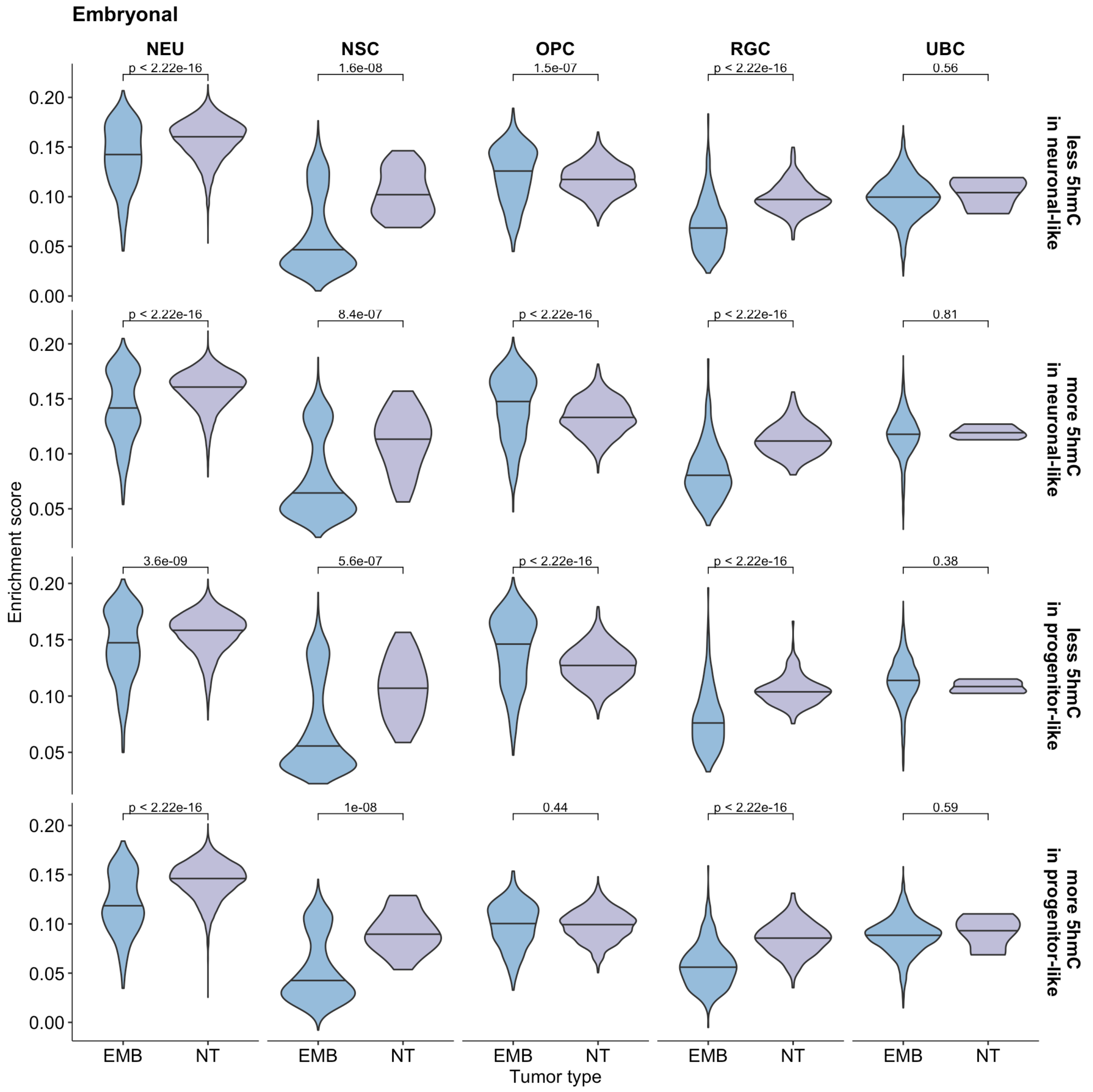

Supplementary Figure 24. Violin plot of enrichment scores of genes with differentially hydroxymethylated CpGs per cell type in embryonal tumors and non-tumor brain tissue. Comparison between tumor and non-tumor was made with two-sided Wilcoxon rank test. Raw p-values are indicated for each comparison.

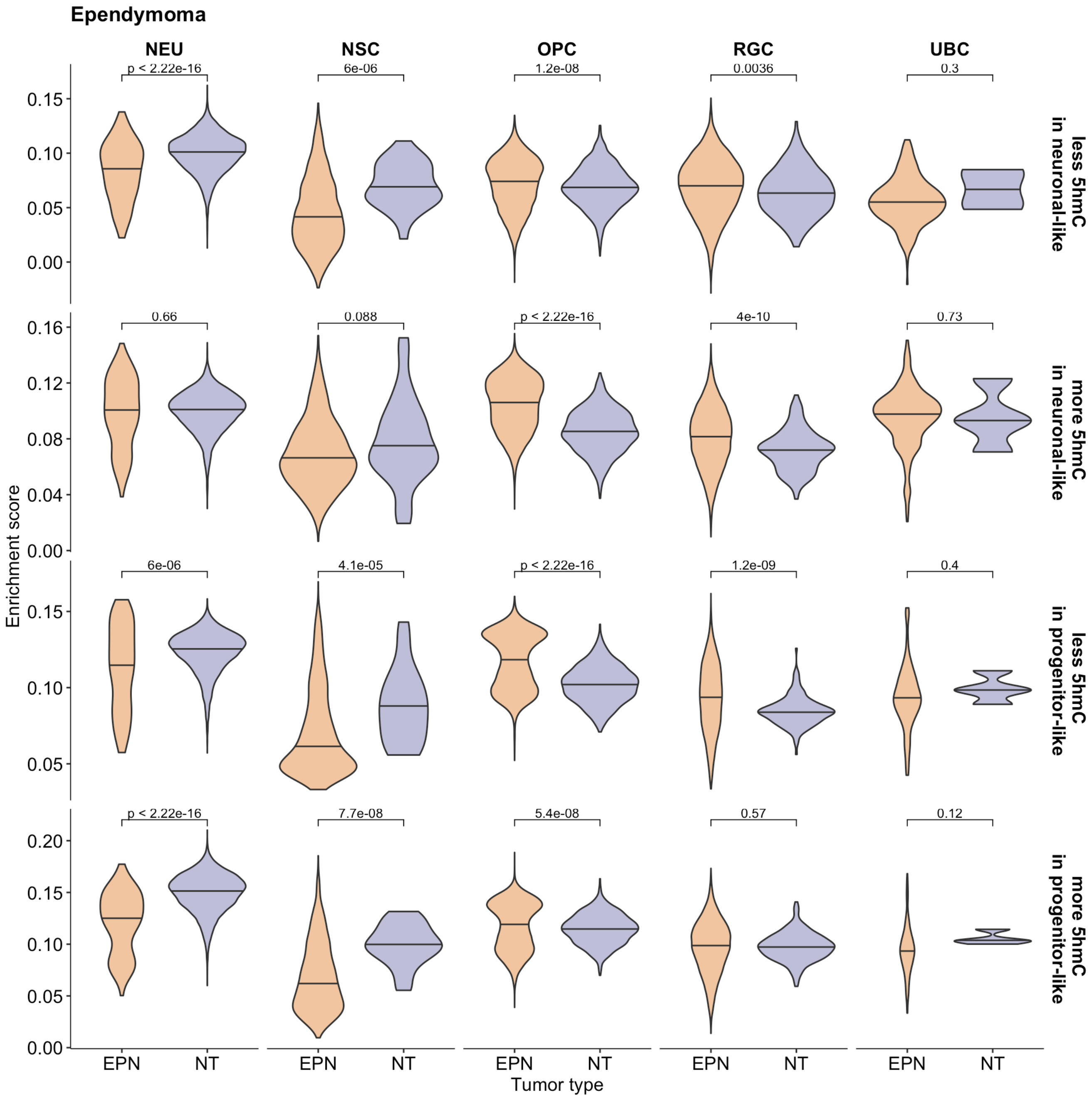

Supplementary Figure 25. Violin plot of enrichment scores of genes with differentially hydroxymethylated CpGs per cell type in ependymoma and non-tumor brain tissue. Comparison between tumor and non-tumor was made with two-sided Wilcoxon rank test. Raw p-values are indicated for each comparison.

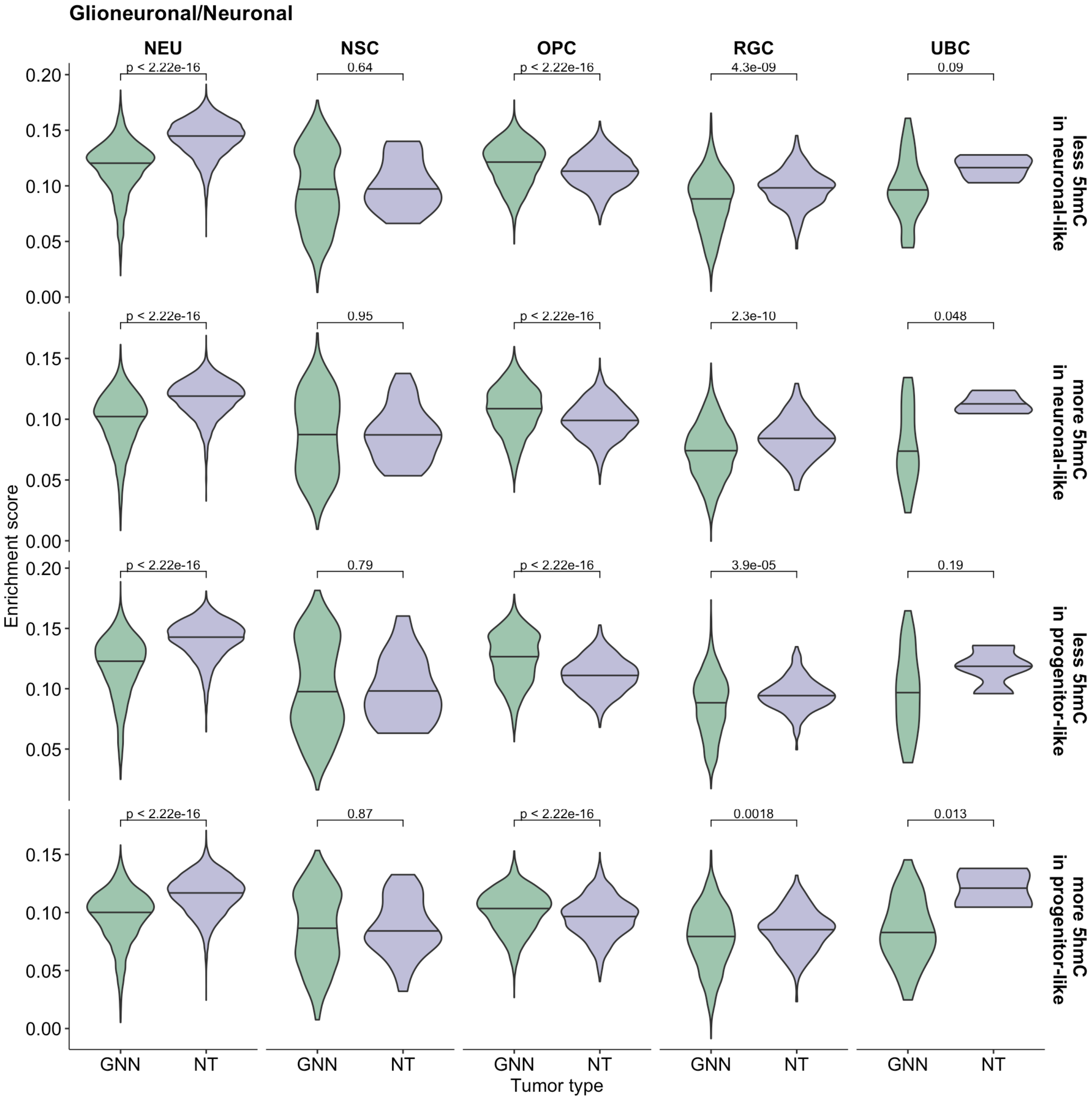

Supplementary Figure 26. Violin plot of enrichment scores of genes with differentially hydroxymethylated CpGs per cell type in glioneuronal/neuronal tumors and non-tumor brain tissue. Comparison between tumor and non-tumor was made with two-sided Wilcoxon rank test. Raw p-values are indicated for each comparison.

	Cytosine modifications	Bulk RNAseq	Single cell RNAseq	Used in manuscript
**Non-tumor**	4	2	3	2
**Astrocytoma**	8	8	8	8
**Embryonal**	6	6	6	6
**Ependymoma**	12	10	12	10
**Glioneuronal/Neuronal**	8	8	8	8
**Total**	38	34	37	34

Supplementary Table 1. Distribution of samples with varying molecular characterization available for analysis.

A

	Estimate	Std Error	P-value
**Genomic context**			
**Promoter**	Referent		
**Gene body**	0.019	0.002	1.44E-14
**Enhancer**	0.030	0.002	<2E-16
**Exon**	0.20	0.002	1.47E-15
**Tumor type**			
**ATC**	Referent		
**EMB**	0.001	0.002	0.66
**EPN**	-0.001	0.002	0.73
**GNN**	-0.002	0.002	0.33

B

	Estimate	Std Error	P-value
**Genomic context**			
**Promoter**	Referent		
**Gene body**	0.038	0.005	1.23E-11
**Enhancer**	0.061	0.005	<2E-16
**Exon**	0.036	0.005	1.62E-10
**Tumor type**			
**ATC**	Referent		
**EMB**	0.041	0.006	1.62E-11
**EPN**	0.021	0.005	3.08E-05
**GNN**	0.005	0.005	0.33

Supplementary Table 2. Association between genomic context, tumor type, grade with **A**) 5-hmC MDI and **B**) 5-mC MDI, determined by linear regression.

A

	Estimate	Std Error	P-value
**Tumor purity**	-0.007	0.005	0.22
**G1**	Referent		
**G2**	0.001	0.004	0.90
**G3**	0.002	0.004	0.68
**G4**	0.0005	0.004	0.90
**NEC/NOS**	0.0004	0.006	0.94

B

	Estimate	Std Error	P-value
**Tumor purity**	0.003	0.012	0.80
**G1**	Referent		
**G2**	0.028	0.010	0.0074
**G3**	0.020	0.008	0.025
**G4**	0.043	0.009	4.5E-5
**NEC/NOS**	0.016	0.013	0.24

Supplementary Table 3. Association between genomic context, tumor type, grade with **A**) 5-hmC MDI and **B**) 5-mC MDI, determined by linear regression.

Measure	Modification	Minimum	Median	Mean	Max	Mean ratio (5-hmC/5-mC)
**Global average**	5-hmC	0.016	0.027	0.032	0.102	0.06
	5-mC	0.448	0.535	0.53	0.579	
**MDI**	5-hmC	0.021	0.038	0.038	0.064	0.494
	5-mC	0.028	0.078	0.077	0.116	

Supplementary Table 4. Summary measures of global averages and MDI of 5-hmC and 5-mC

ProbeID	Type	UCSC_RefGene_Name	Regulatory_Feature_Group	Relation_to_Island	TSS200	TSS1500	Body	Exon1	UTR5	UTR3	ExonBnd	Phantom5_Enhancer	DHS	OpenChromatin	TFBS
**cg00256046**	II	ESRRG		N_Shore	0	0	0	0	1	0	0	0	1	0	0
**cg00519299**	II	GPR44		S_Shelf	1	0	0	0	0	0	0	0	1	0	0
**cg01230224**	II		Unclassified	OpenSea	0	0	0	0	0	0	0	0	0	0	1
**cg01993818**	I	NPAS3;NPAS3;NPAS3;NPAS3		Island	0	0	0	0	0	1	0	0	1	0	0
**cg02222170**	II			OpenSea	0	0	0	0	0	0	0	0	1	0	0
**cg02559101**	II	MIR1273E		OpenSea	0	0	1	0	0	0	0	1	0	0	0
**cg03823494**	II	SIX5;DMPK;DMPK;DMPK;DMPK;DMPK;DMPK;DMPK	Promoter_Associated	Island	0	1	0	0	0	1	0	0	1	0	0
**cg04511352**	II			OpenSea	0	0	0	0	0	0	0	0	0	0	1
**cg04864309**	II			OpenSea	0	0	0	0	0	0	0	0	0	0	0
**cg05168012**	I		Unclassified_Cell_type_specific	Island	0	0	0	0	0	0	0	0	0	1	0
**cg08089031**	I	HECA	Promoter_Associated	Island	1	0	0	0	0	0	0	0	1	0	0
**cg08278401**	II	LRRC72		N_Shore	0	1	0	0	0	0	0	0	1	0	0
**cg08354093**	I	THBD		Island	0	0	0	1	0	0	0	0	1	0	0
**cg08412913**	I			OpenSea	0	0	0	0	0	0	0	0	1	0	0
**cg09211399**	II		Unclassified	Island	0	0	0	0	0	0	0	0	1	0	0
**cg09346823**	II			OpenSea	0	0	0	0	0	0	0	0	0	0	0
**cg10653504**	I	ATP8B3;ATP8B3;ATP8B3	Promoter_Associated	Island	0	0	1	0	1	0	0	0	1	0	0
**cg13218435**	II	PET112L	Promoter_Associated	S_Shore	0	1	0	0	0	0	0	0	0	1	1
**cg13873632**	II	LDB2;LDB2		OpenSea	0	0	1	0	0	0	0	0	1	0	0
**cg13924635**	I	SDF4;SDF4;B3GALT6		Island	0	1	0	1	0	0	0	0	1	0	0
**cg14835472**	I	THBD		Island	1	0	0	0	0	0	0	0	1	0	0
**cg15219899**	II			OpenSea	0	0	0	0	0	0	0	0	1	0	0
**cg17689498**	II	TJP1;TJP1;TJP1;TJP1		OpenSea	0	0	1	0	0	0	0	0	0	0	0
**cg18280362**	I	CWH43		Island	1	0	0	0	0	0	0	0	1	0	0
**cg20843075**	II			OpenSea	0	0	0	0	0	0	0	0	0	0	1
**cg22532674**	II	THBD	Unclassified_Cell_type_specific	Island	0	1	0	0	0	0	0	0	1	0	0
**cg23529010**	II	CYP4A11		OpenSea	0	1	0	0	0	0	0	0	0	0	0
**cg26395382**	I	C10orf116;C10orf116		Island	0	0	0	1	1	0	0	0	1	0	0

Supplementary Table 5. Annotation of gene name and genomic context of 28 dhmCpGs shared across tumor types

Context	Proportion
Island	0.43
N_Shore	0.07
OpenSea	0.43
S_Shelf	0.04
S_Shore	0.04

Supplementary Table 6. Proportion of shared dhmCpGs relative to CpG islands.

Context	Proportion
TSS200	0.14
TSS1500	0.21
Body	0.14
1st exon	0.11
UTR5	0.11
UTR3	0.07
Exon bound	0
Phantom5 Enhancer	0.04
DHS	0.64
OpenChromatin	0.07
TFBS	0.14

Supplementary Table 7. Proportion of shared dhmCpGs in different genomic contexts.

Astrocytoma					
	**Body**	**Both**	**Neither**	**Promoter**	**Total**
**Decrease**	0	0	0	0	0
**Increase**	5	0	4	3	12
**Total**	5	0	4	3	12
**Embryonal tumors**					
	**Body**	**Both**	**Neither**	**Promoter**	**Total**
**Decrease**	6	0	1	10	17
**Increase**	44	0	13	17	74
**Total**	50	0	14	27	91
**Ependymoma**					
	**Body**	**Both**	**Neither**	**Promoter**	**Total**
**Decrease**	0	0	0	1	1
**Increase**	1	0	0	4	5
**Total**	1	0	0	5	6
**Glioneuronal/neuronal tumors**					
	**Body**	**Both**	**Neither**	**Promoter**	**Total**
**Decrease**	0	0	0	0	0
**Increase**	6	0	2	5	13
**Total**	6	0	2	5	13

Supplementary Table 8. The number of differentially expressed genes with differentially hydroxymethylated CpGs identified by bulk tissue epigenome-wide association study. Categorized by the direction of gene expression change and the genomic context of the differentially hydroxymethylated CpGs for each tumor type.

Tumor type	Nuclei N	Neuronal-like	Progenitor-like	NEU	NSC	OPC	RGC	UBC
**Astrocytoma**	7714	1280	4543	1280	685	3269	485	104
**Embryonal**	12936	462	7649	462	3380	446	448	3375
**Ependymoma**	22287	204	19679	204	1695	9582	8120	282
**Glioneuronal/ Neuronal**	16016	3848	4694	3848	731	2189	1730	44
**Non-Tumor**	17451	8394	1431	8394	29	1224	174	4

Supplementary Table 9. The number of nuclei from single nuclei RNA-seq that were included in the CellDMC analysis.

	ATC	EMB	EPN	GNN
**Hypo-hydroxymethylated in neuronal-like cells**	3741	4829	1031	2963
**Hyper-hydroxymethylated in neuronal-like cells**	15892	6589	2161	2027
**Hypo-hydroxymethylated in progenitor-like cells**	2270	16099	40216	5233
**Hyper-hydroxymethylated in progenitor-like cells**	2270	2644	4111	1444

Supplementary Table 10. The number of differentially hydroxymethylated CpGs per tumor type identified by CellDMC. Categorized by the change in the level of hydroxymethylation and cell type of association.
