# Supplementary material for: Associations in cell type-specific hydroxymethylation and transcriptional alterations of pediatric central nervous system tumors
Source: Nat Commun. 2024 Apr 30;15:3635. doi: 10.1038/s41467-024-47943-9 (PMC11061294; doi:10.1038/s41467-024-47943-9)
Supplement: Supplementary file 3 — Description of Additional Supplementary Files [file 41467_2024_47943_MOESM3_ESM.pdf]

Description of Additional Supplementary Files

Supplementary Data 1. Statistically significant results from epigenome wide association study comparing astrocytoma to non-tumor tissue in the unadjusted model.

Supplementary Data 2. Statistically significant results from epigenome wide association study comparing astrocytoma to non-tumor tissue in the adjusted model including all five cell types.

Supplementary Data 3. Statistically significant results from epigenome wide association study comparing embryonal tumors to non-tumor tissue in the unadjusted model.

Supplementary Data 4. Statistically significant results from epigenome wide association study comparing embryonal tumors to non-tumor tissue in the adjusted model including all five cell types.

Supplementary Data 5. Statistically significant results from epigenome wide association study comparing ependymoma to non-tumor tissue in the unadjusted model.

Supplementary Data 6. Statistically significant results from epigenome wide association study comparing ependymoma to non-tumor tissue in the adjusted model including all five cell types.

Supplementary Data 7. Statistically significant results from epigenome wide association study comparing glioneuronal/neuronal tumors to non-tumor tissue in the unadjusted model.

Supplementary Data 8. Statistically significant results from epigenome wide association study comparing glioneuronal/neuronal tumors to non-tumor tissue in the adjusted model including all five cell types.

Supplementary Data 9. Results from differential gene expression analysis comparing astrocytoma to non-tumor brain tissue in the unadjusted model.

Supplementary Data 10. Results from differential gene expression analysis comparing astrocytoma to non-tumor brain tissue in the adjusted for cell type proportions and tumor purity.

Supplementary Data 11. Results from differential gene expression analysis comparing embryonal tumors to non-tumor brain tissue in the unadjusted model.

Supplementary Data 12. Results from differential gene expression analysis comparing embryonal tumors to non-tumor brain tissue in the adjusted for cell type proportions and tumor purity.

Supplementary Data 13. Results from differential gene expression analysis comparing ependymoma to non-tumor brain tissue in the unadjusted model.

Supplementary Data 14. Results from differential gene expression analysis comparing ependymoma to non-tumor brain tissue in the adjusted for cell type proportions and tumor purity.

Supplementary Data 15. Results from differential gene expression analysis comparing glioneuronal/neuronal tumors to non-tumor brain tissue in the unadjusted model.

Supplementary Data 16. Results from differential gene expression analysis comparing glioneuronal/neuronal tumors to non-tumor brain tissue in the adjusted for cell type proportions and tumor purity.

Supplementary Data 17. Results from cell type proportion adjusted epigenome wide association study with CellDMC comparing astrocytoma to non-tumor brain tissue.

Supplementary Data 18. Results from cell type proportion adjusted epigenome wide association study with CellDMC comparing embryonal tumors to non-tumor brain tissue.

Supplementary Data 19. Results from cell type proportion adjusted epigenome wide association study with CellDMC comparing ependymoma to non-tumor brain tissue.

Supplementary Data 20. Results from cell type proportion adjusted epigenome wide association study with CellDMC comparing glioneuronal/neuronal tumors to non-tumor brain tissue.

Supplementary Data 21. Cell type-driven dhmCpGs and dmCpGs enrichment at genomic contexts. Odds ratio and 95% confidence intervals calculated by the Mantel-Haenszel test.
